# The transcription factor EGR2 is indispensable for tissue-specific imprinting of alveolar macrophages in health and tissue repair^#^

**DOI:** 10.1126/sciimmunol.abj2132

**Published:** 2021-11-19

**Authors:** Jack McCowan, Frédéric Fercoq, Phoebe M. Kirkwood, Wouter T’Jonck, Lizi M. Hegarty, Connar M. Mawer, Richard Cunningham, Ananda S. Mirchandani, Anna Hoy, Duncan C. Humphries, Gareth-Rhys Jones, Carsten G. Hansen, Nik Hirani, Stephen J. Jenkins, Sandrine Henri, Bernard Malissen, Sarah R. Walmsley, David H. Dockrell, Philippa T. K. Saunders, Leo M. Carlin, Calum C. Bain

**Affiliations:** 1 University of Edinburgh Centre for Inflammation Research, Queens Medical Research Institute, 47 Little France Crescent, Edinburgh BioQuarter, Edinburgh, EH16 4TJ, UK; 2 Institute for Regeneration and Repair, University of Edinburgh, 5 Little France Drive, Edinburgh BioQuarter, Edinburgh EH16 4UU, UK; 3 Cancer Research UK Beatson Institute, Glasgow, G61 1BD, UK; 5 Institute of Immunology & Infection Research, School of Biological Sciences, University of Edinburgh, Edinburgh EH9 3FL, UK; 7 Centre d'Immunologie de Marseille-Luminy, Aix Marseille Université UM2, INSERM, U1104, CNRS UMR7280, 13288 Marseille, France; 8 Institute of Cancer Sciences, University of Glasgow, Glasgow, G61 1QH, UK

## Abstract

Alveolar macrophages are the most abundant macrophages in the healthy lung where they play key roles in homeostasis and immune surveillance against air-borne pathogens. Tissue-specific differentiation and survival of alveolar macrophages relies on niche-derived factors, such as granulocyte-macrophage colony stimulating factor 2 (GM-CSF) and transforming growth factor beta (TGF-β). However, the nature of the downstream molecular pathways that regulate the identity and function of alveolar macrophages and their response to injury remains poorly understood. Here, we identify that the transcription factor EGR2 is an evolutionarily conserved feature of lung alveolar macrophages and show that cell-intrinsic EGR2 is indispensable for the tissue-specific identity of alveolar macrophages. Mechanistically, we show that EGR2 is driven by TGF-β and GM-CSF in a PPAR-γ-dependent manner to control alveolar macrophage differentiation. Functionally, EGR2 was dispensable for regulation of lipids in the airways, but crucial for the effective handling of the respiratory pathogen *Streptococcus pneumoniae*. Finally, we show that EGR2 is required for repopulation of the alveolar niche following sterile, bleomycin-induced lung injury and demonstrate that EGR2-dependent, monocyte-derived alveolar macrophages are vital for effective tissue repair following injury. Collectively, we demonstrate that EGR2 is an indispensable component of the transcriptional network controlling the identity and function of alveolar macrophages in health and disease.

## Introduction

Alveolar macrophages provide a first line of defence against airborne pathogens, as well as maintaining lung homeostasis and orchestrating tissue repair following injury. However, in chronic lung pathologies such as allergic asthma, idiopathic pulmonary fibrosis (IPF) and chronic obstructive pulmonary disease (COPD), alveolar macrophages display aberrant activity and, in many cases, appear to perpetuate disease (1). Moreover, monocytes and macrophages appear to play a particular pathogenic role in the context of severe coronavirus disease 2019 (COVID-19) (2–4). Thus, understanding the environmental signals and downstream molecular pathways that control the tissue-specific imprinting of macrophages in different contexts may yield important insights into how lung-specific cues regulate homeostasis and susceptibility to disease.

Alveolar macrophages are derived from foetal progenitors that seed the lung during embryonic development (5–7). However, the characteristic phenotype and functional properties of alveolar macrophages do not develop until the first few days of postnatal life in parallel with alveolarisation of the lung and are controlled by GM-CSF (also known as CSF-2) (7, 8) and the immunoregulatory cytokine TGF-β (9). Together these cytokines induce expression of the transcription factor peroxisome proliferator-activated receptor gamma (PPAR-γ) to promote survival and tissue-specific specialisation, including upregulation of genes involved in lipid uptake and metabolism (8). Consequently, mice in which *Csf2rb, Tgfbr2* or *Pparg* has been genetically ablated in myeloid cells develop spontaneous pulmonary alveolar proteinosis (8, 9). However, alveolar macrophages largely fail to develop in the absence of GM-CSF and TGF-β receptor signalling due to their key role in macrophage survival. Therefore, it remains unclear if or how these factors control the tissue-specific identity and function of alveolar macrophages. Moreover, while considered the ‘master transcription factor’ of alveolar macrophages, PPAR-γ has been implicated in the control of other tissue macrophages, including splenic red pulp macrophages (10, 11), and thus, the transcriptional network responsible for conferring specificity upon alveolar macrophage differentiation remains unclear. Finally, if and how additional transcriptional regulators are involved in regulating these processes in the context of inflammation and repair is largely unexplored.

Here, we have used single cell RNA sequencing (scRNA-seq) to identify the transcriptional regulators expressed by alveolar macrophages. We show that expression of the transcription factor EGR2 is a distinct feature of lung alveolar macrophages. Using cell-specific ablation of *Egr2* and mixed bone marrow chimeric mice, we show that cell-intrinsic EGR2 is indispensable for the tissue-specific identity of alveolar macrophages and their ability to control infection with a major respiratory pathogen, *Streptococcus pneumoniae*. RNA sequencing (RNA-seq) shows that EGR2 controls a large proportion of the core transcriptional signature of alveolar macrophages, including expression of *Siglec5, Epcam* and *Car4*. Mechanistically, we show that EGR2 expression is induced by TGF-β and GM-CSF-dependent signalling, and acts to maintain expression of CCAAT-enhancer-binding protein beta (C/EBP-β) to control alveolar macrophage differentiation. Finally, using the bleomycin-induced model of lung injury and a combination of fate mapping approaches, we show that post-injury repopulation of the alveolar macrophage niche occurs via differentiation of bone marrow-derived cells in an EGR2-dependent manner and that these monocyte-derived macrophages are indispensable for effective tissue repair and resetting of tissue homeostasis.

## Results

### EGR2 expression is a selective property of alveolar macrophages

**Figure 1:**
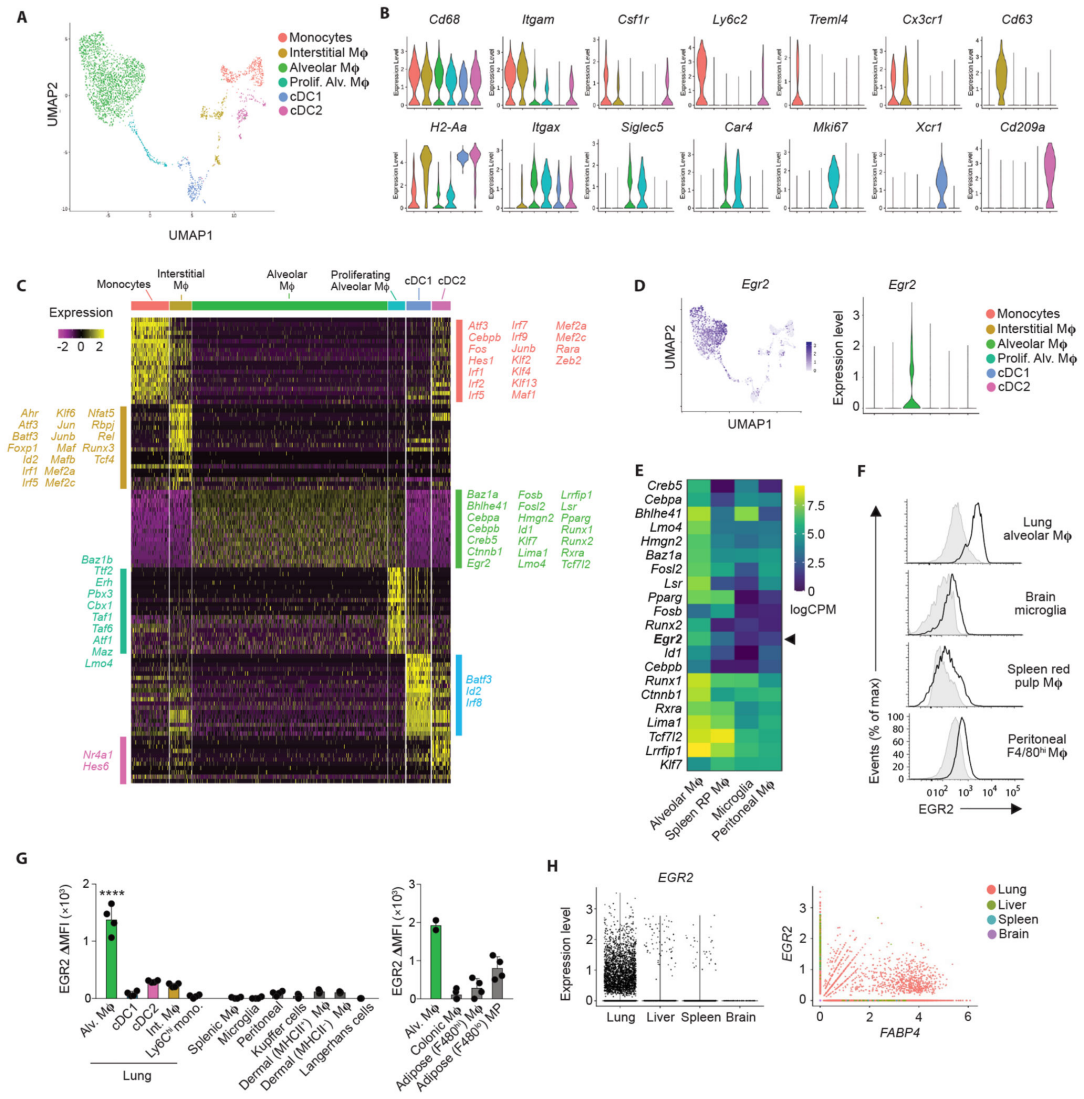
EGR2 expression is a selective property of alveolar macrophages **A.** UMAP dimensionality reduction analysis of 3936 cells (non-granulocyte, myeloid cells) reveals six clusters of mononuclear phagocytes in murine lungs. Cells obtained from an individual *Rag1* ^–/–^ mouse. **B.** Feature plots displaying expression of individual genes by clusters identified in **A.** **C.** Heatmap showing the top 20 most differentially expressed genes by each cluster defined in **A**. and annotated to show upregulated transcription factors/regulators within each cluster. **D.** Overlay UMAP plot and feature plot showing expression of *Egr2* by clusters identified in **A.** **E.** Heatmap showing relative expression of selected transcription factors by lung alveolar macrophages, CD102^+^ peritoneal macrophages, brain microglia and red pulp splenic macrophages as derived from the ImmGen consortium. **F.** Representative expression of EGR2 by lung alveolar macrophages, CD102^+^ peritoneal macrophages, brain microglia and red pulp splenic macrophages obtained from adult unmanipulated C57BL/6 mice. Shaded histograms represent isotype controls. Data are from one of three independent experiments. **G.** Expression of EGR2 by the indicated macrophage and myeloid cell populations shown as relative MFI (MFI in *Egr2* ^fl/fl^ – MFI in *Lyz2* ^Cre/+^.*Egr2* ^fl/fl^ mice). Adipose & colonic macrophages shown on a separate graph due to measurements performed in an independent experiment with different flow cytometer settings. Repeat data for alveolar macrophages included as a reference. Data represent 3-4 mice (*left graph*) or 2-4 mice (right graph) per tissue. **** p<0.0001 (One-way ANOVA followed by Tukey’s multiple comparisons post-test). **H.** *In silico* analysis of *EGR2* and *FABP4* expression by lung, liver, spleen and brain macrophages extracted on the basis of *C1QA^+^* expression from (17–19).

To begin to dissect the molecular pathways underlying the niche-specific imprinting of alveolar macrophages, we performed scRNA-seq of murine lung mononuclear phagocytes from lung digests to identify the transcriptional profile of alveolar macrophages. To this end, non-granulocytic CD45^+^ cells from lungs of *Rag1* ^–/–^ mice were purified by FACS and sequenced using the 10x Chromium platform (Supplementary Figure 1A). *Rag1* ^–/–^ mice were used to enrich for myeloid cells and reduce potential contamination by lymphocyte-macrophage doublets. 3936 cells passed quality control and were clustered using Uniform Manifold Approximation and Projection (UMAP) dimensionality reduction analysis within the *Seurat* R package. NK cells, identified by their expression of *Ncr1, Nkg7* and *Gzma*, were excluded (Supplementary Figure 1A, B) and the remaining myeloid cells were re-clustered to leave six clusters of mononuclear phagocytes, and these were annotated using known landmark gene expression profiles (Figure 1A, B). Cluster 1 represented monocytes based on their expression of *Itgam* (encoding CD11b)*, Csf1r* and *Cd68*, and could be divided into classical and non-classical monocytes based on expression of *Ly6c2* and *Treml4* respectively (Figure 1A, B). Cluster 2 represented interstitial macrophages based on their high expression of *Cx3cr1*, *Cd68, Csf1r* and *H2-Aa* and lack of the *Xcr1* and *Cd209a* genes which defined cDC1 (cluster 5) and cDC2 (cluster 6) respectively. Alveolar macrophages (cluster 3) formed the largest population and could be defined by their expression of *Itgax* (encoding CD11c)*, Siglec5* (encoding SiglecF) and *Car4*, and lack of *Cx3cr1* and *Itgam*. Cluster 4 was transcriptionally similar to cluster 3, but was defined by genes associated with cell cycle, including *Mki67, Birc5* and *Tubb5*, suggesting these represent proliferating alveolar macrophages (Figure 1A, B).

Next, we compared gene expression profiles of these clusters, focussing on genes more highly expressed by alveolar macrophages relative to all other mononuclear phagocytes. 722 genes fitted these criteria, including *Fapb1*, *Spp1* (encoding osteopontin) and *Cidec* which are known to be specifically and highly expressed by alveolar macrophages (Data File S1) (12, 13). Within this cassette of genes, we turned our attention to genes encoding transcription factors/regulators, as we hypothesised that these might control the tissue specific differentiation of alveolar macrophages. As expected, these included *Pparg, Cebpb* and *Bhlhe41* which have been shown to control the development and self-renewal capacity of alveolar macrophages (8) (10, 14–16) (Figure 1C). However, this analysis also revealed transcription factors such as *Id1, Klf7* and *Egr2* which have not previously been implicated in the control of alveolar macrophage differentiation. We focussed on EGR2, which is part of a family of early growth response (EGR) transcription factors, comprising EGR1-4, as *Egr2* appeared to be expressed in a particularly selective manner by alveolar macrophages (Figure 1D) when compared with other tissue macrophages at mRNA (Figure 1E) and protein level (Figure 1F, G & Supplementary Figure 2A). In contrast, while highly expressed by alveolar macrophages, *Pparg* was also expressed at a high level by splenic red pulp macrophages (Figure 1E), consistent with previous reports (10, 11). Of note, we did detect moderate EGR2 expression in F4/80^lo^ mononuclear phagocytes in adipose tissue, whereas F4/80^hi^ macrophage had low levels of EGR2 (Figure 1G). While our scRNA-seq analysis suggested that *Egr2* was expressed at lower levels by proliferating alveolar macrophages, we could not confirm this at protein level, with Ki67^+^ alveolar expressing equivalent levels of EGR2 to their Ki67^–^ counterparts (Supplementary Figure 2B). Next, we performed analogous analysis of *EGR2* expression across a variety of human macrophage populations from scRNA-seq data sets within the Human Cell Atlas (17–19). Consistent with our analysis in the mouse, this showed that *EGR2* expression was confined to lung macrophages, and in particular *FABP4* ^+^ macrophages which correspond to airway macrophages (Figure 1H), and we confirmed this at protein level, showing that human CD163^+^HLA-DR^+^ bronchoalveolar lavage (BAL) macrophages uniformly express EGR2 (Supplementary Figure 2C). Thus, these data demonstrate that EGR2 expression is a constitutive, specific and evolutionarily conserved feature of alveolar macrophages.

### EGR2 is required for the phenotypic identity of alveolar macrophages

**Figure 2:**
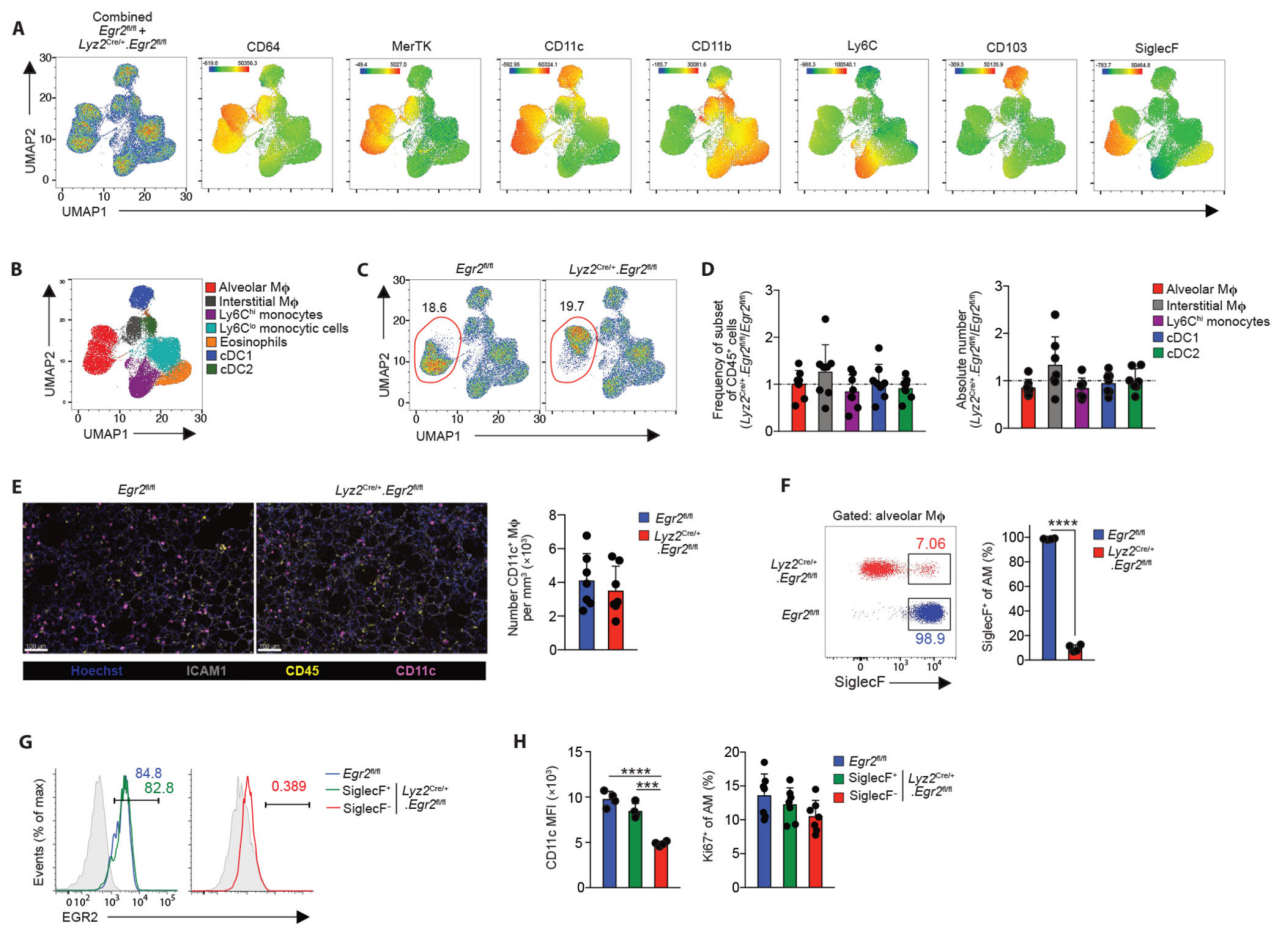
EGR2 is required for the phenotypic identity of alveolar macrophages **A.** UMAP analysis of CD3^–^CD19^–^NK1.1^–^Ly6G^–^CD11b^+^/CD11c^+^ cells pooled from adult unmanipulated *Egr2* ^fl/fl^ and *Lyz2* ^Cre/+^.*Egr2* ^fl/fl^ mice (*left panel*). Heatmap plots showing the relative expression of the indicated markers by myeloid clusters. **B.** Cluster identity confirmed by manual gating (see Fig. S3A). **C.** Relative frequency of alveolar macrophages of all CD45^+^ leukocytes in unmanipulated adult *Egr2* ^fl/fl^ and *Lyz2* ^Cre/+^.*Egr2* ^fl/fl^ mice. **D.** Relative frequency and absolute number of alveolar macrophages, cDC1, cDC2, Ly6C^hi^ monocytes and CD64^+^MHCII^+^ interstitial macrophages in lung digests from adult unmanipulated *Lyz2* ^Cre/+^.*Egr2* ^fl/fl^ mice compared with their abundance in *Egr2* ^fl/fl^ littermates. Data are pooled from three independent experiments with 8 mice per group. **E.** Confocal fluorescence imaging of Fixed Precision Cut Lung Slices from adult unmanipulated *Egr2* ^fl/fl^ or *Lyz2* ^Cre/+^.*Egr2* ^fl/fl^ mice stained with antibodies against ICAM1, CD45 and CD11c (*left*) and quantification of the number of CD11c^+^ macrophages per mm^3^ in each group (*right*). Data are pooled from three independent experiments with 7 mice per group. **F.** Representative expression of SiglecF by CD11c^hi^CD11b^lo^ alveolar macrophages (from **F**) obtained from lung digests from adult unmanipulated *Egr2* ^fl/fl^ or *Lyz2* ^Cre/+^.*Egr2* ^fl/fl^ mice (*left*), frequency of SiglecF^+^ macrophages in each strain (*right*). Data are from 4 mice per group from one of at least 5 independent experiments. SiglecF; ****p<0.0001 (unpaired Student’s *t*-test). **G.** Representative expression of EGR2 by SiglecF-defined alveolar macrophages. Shaded histograms represent isotype controls. Data are from one of three independent experiments. **H.** Mean fluorescence intensity (MFI) of CD11c expression (*left*) and frequency of Ki67^+^ SiglecF-defined CD11c^hi^CD11b^lo^ alveolar macrophages (*right*) amongst lung digests from adult unmanipulated *Egr2* ^fl/fl^ and *Lyz2* ^Cre/+^.*Egr2* ^fl/fl^ mice. Data are from 4 mice per group from one of at least 5 independent experiments. *** p<0.001, **** p<0.0001 (One-way ANOVA followed by Tukey’s multiple comparisons post-test). Symbols represent individual mice in all graphs and error bars represent the standard deviation.

Previous work has suggested that EGR1 and EGR2 act in a redundant manner (20), while other studies have suggested EGR transcription factors are completely dispensable for macrophage differentiation (21). However, many of these studies were performed *in vitro* and the roles of EGRs in tissue-specific macrophage differentiation has not been assessed comprehensively *in vivo*, in part, due to the postnatal lethality of global *Egr2^–/–^* mice (22, 23). To determine the role of EGR2 in alveolar macrophage development and differentiation, we crossed *Lyz2* ^Cre^ mice (24) with *Egr2* ^fl/fl^ mice (25), to generate a strain in which myeloid cells, including monocytes, macrophages, dendritic cells and neutrophils, lack EGR2 in a constitutive manner. We performed unbiased UMAP flow cytometry analysis on lung leukocytes obtained from *Lyz2* ^Cre^.*Egr2* ^fl/fl^ mice and *Egr2* ^fl/fl^ littermate controls, focussing on ‘lineage’ negative (CD3^–^CD19^–^ NK1.1^–^Ly6G^–^) CD11c^+^ and CD11b^+^ cells in lung digests (Figure 2A). Surface marker analysis of cells pooled from *Egr2* ^fl/fl^ and *Lyz2* ^Cre/+^.*Egr2* ^fl/fl^ mice confirmed the presence of alveolar and interstitial macrophages, eosinophils and subsets of dendritic cells and monocytes (Figure 2A) and this was validated by manual gating (Figure 2B & Supplementary Figure 3A). Due to their CD11c^hi^CD11b^–^ phenotype, alveolar macrophages clustered separately from the other CD11b^+^ myeloid cells (Figure 2A-C). All myeloid cells, including alveolar macrophages, were equally abundant in the lungs of *Egr2* ^fl/fl^ and *Lyz2* ^Cre/+^.*Egr2* ^fl/fl^ mice (Figure 2D, E). However, whereas alveolar macrophages from *Egr2* ^fl/fl^ mice expressed high levels of SiglecF, the majority of alveolar macrophages obtained from *Lyz2* ^Cre/+^.*Egr2* ^fl/fl^ mice lacked SiglecF expression (Figure 2F), explaining their distinct positioning within the alveolar macrophage cluster in the UMAP analysis. Indeed only ~5% of alveolar macrophages in *Lyz2* ^Cre/+^.*Egr2* ^fl/fl^ mice expressed high levels of SiglecF, and further analysis showed that these expressed high levels of EGR2 (Figure 2G), suggesting that the SiglecF^+^ cells remaining in the *Lyz2* ^Cre/+^.*Egr2* ^fl/fl^ mouse represent cells that have escaped Cre-mediated recombination. Consistent with this, SiglecF^+^ cells in the *Lyz2* ^Cre/+^.*Egr2* ^fl/fl^ mouse expressed high levels of CD11c equivalent to alveolar macrophages from control mice, whereas SiglecF^–^ alveolar macrophages expressed lower levels of CD11c (Figure 2H). We did not detect differences in the proliferative activity of *Egr2*-sufficient and -deficient alveolar macrophages (Figure 2H). Importantly and consistent with the lack of EGR2 expression by other tissue resident macrophages, we saw no effect on the cell number and expression of signature markers by resident macrophages in other tissues, including in the spleen where macrophages share a dependence on PPAR-γ (10, 11) and adipose tissue where we detected EGR2 expression (Supplementary Figure 3B-D). Thus, these data demonstrate that while EGR2 expression is dispensable for alveolar macrophages survival and self-maintenance, it is indispensable for imprinting key phenotypic features of the cells in the healthy lung.

### EGR2 controls the tissue-specific transcriptional programme of alveolar macrophages

**Figure 3:**
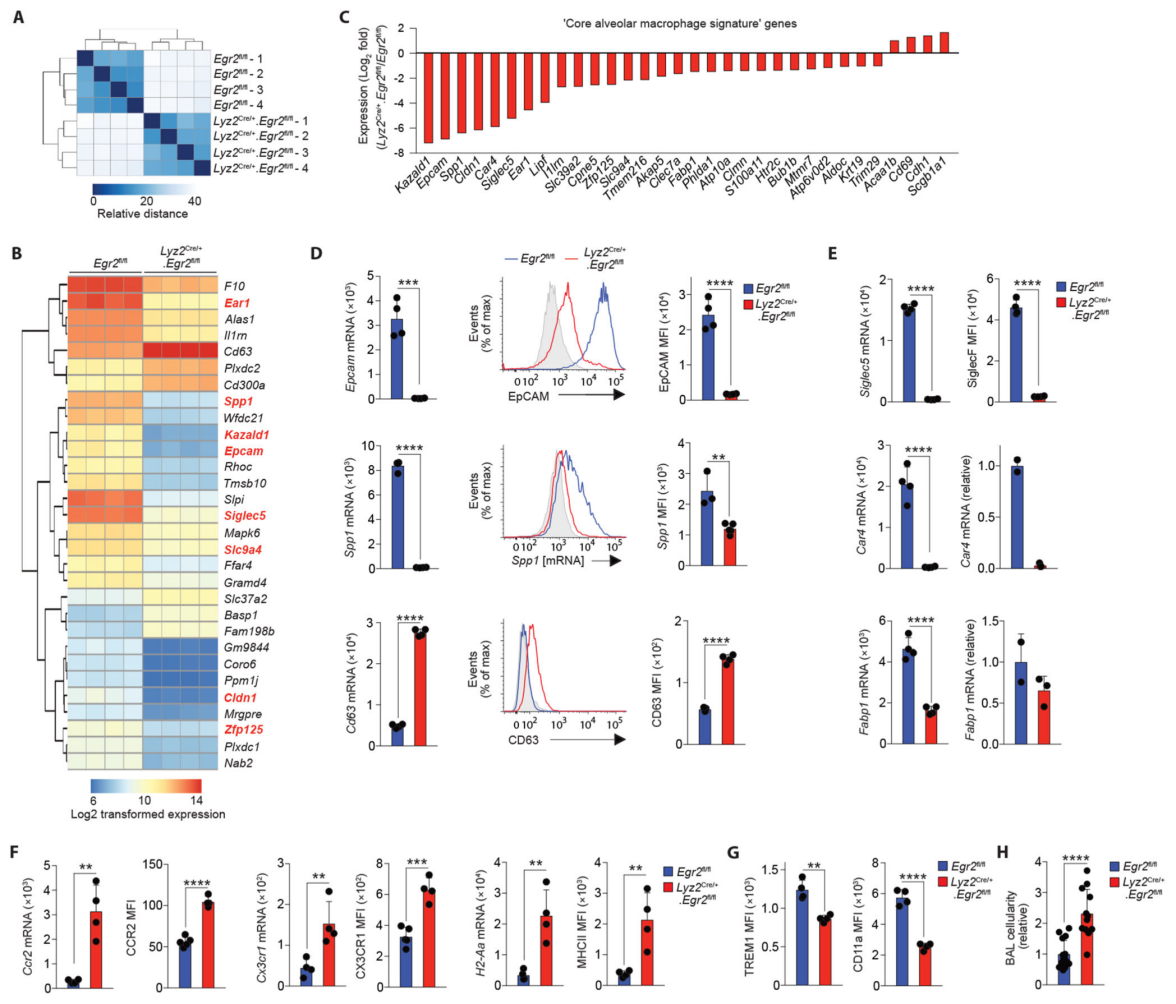
EGR2 controls tissue-specific transcriptional programme of alveolar macrophages **A.** Heatmap of RNA-seq data showing the euclidean distance between samples from adult *Egr2* ^fl/fl^ and *Lyz2* ^Cre/+^.*Egr2* ^fl/fl^ mice. **B.** Heatmap showing log2 transformed expression of the 30 most differentially expressed genes by alveolar macrophages *Egr2* ^fl/fl^ and *Lyz2* ^Cre/+^.*Egr2* ^fl/fl^ mice. Each column represents a biological replicate with four mice per group. Genes highlighted in red appear in the ‘core signature’ of alveolar macrophages as defined by the ImmGen Consortium (12). **C.** Log_2_-fold expression of differentially expressed genes that form part of the ‘core signature’ of alveolar macrophages as defined by the ImmGen Consortium (12). **D.** Expression of *Epcam*, *Spp1* and *Cd63* from the RNA-seq dataset (*left panels*), representative flow cytometric validation of EpCAM, *Spp1* (mRNA detected by PrimeFlow technology) and CD63 expression (*middle panels*) and replicate MFI expression data of each of these markers by alveolar macrophages from adult unmanipulated *Egr2* ^fl/fl^ and *Lyz2* ^Cre/+^.*Egr2* ^fl/fl^ mice. Data are from one of two independent experiments with 5 (Cre^–^) and 4 (Cre^+^) mice per group. **E.** Expression of *Siglec5*, *Car4* and *Fabp1* from the RNA-seq dataset (*left panels*) and validation by flow cytometry (SiglecF) or qPCR (*Car4*, *Fabp1*). Data for SiglecF is from one of at least 10 independent experiments with 5 (*Egr2* ^fl/fl^) and 4 (*Lyz2* ^Cre/+^.*Egr2* ^fl/fl^) mice per group. Data for *Car4* and *Fabp1* represents 2 (*Egr2* ^fl/fl^) and 4 (*Lyz2* ^Cre/+^.*Egr2* ^fl/fl^) mice per group. **F.** Expression of *Ccr2*, *Cx3cr1* and *H2-Aa* from the RNA-seq dataset and replicate MFI expression data of CCR2, CX3CR1 and MHCII as determined by flow cytometry. Data are from one of two independent experiments with 5 (*Egr2* ^fl/fl^) and 4 (*Lyz2* ^Cre/+^.*Egr2* ^fl/fl^) mice per group. **G.** Replicate MFI data of for CD11a and TREM1 expression as determined by flow cytometry. Data are from one of two independent experiments with 5 (*Egr2* ^fl/fl^) and 4 (*Lyz2* ^Cre/+^.*Egr2* ^fl/fl^) mice per group. **H.** Absolute number of CD11c^hi^CD11b^–^ alveolar macrophages present in the BAL of adult unmanipulated *Lyz2* ^Cre/+^.*Egr2* ^fl/fl^ mice relative to their abundance in *Egr2* ^fl/fl^ littermates. Data are pooled from three independent experiments with 15 (*Egr2* ^fl/fl^) and 12 (*Lyz2* ^Cre/+^.*Egr2* ^fl/fl^) mice per group. Symbols represent individual mice in all graphs and error bars represent the standard deviation. *p<0.05, **p<0.01, ***p<0.001, ****p<0.0001 (unpaired Student’s *t* test).

The failure of alveolar macrophages from *Lyz2* ^Cre/+^.*Egr2* ^fl/fl^ mice to express SiglecF suggested that the tissue-specific differentiation programme of these cells may be altered by *Egr2* deficiency. Hence, to ascertain the global effects of *Egr2* deletion on alveolar macrophage differentiation, we next performed bulk RNA-seq of CD11c^hi^CD11b^lo^ alveolar macrophages from lung digests of *Egr2* ^fl/fl^ and *Lyz2* ^Cre/+^.*Egr2* ^fl/fl^ mice (using only SiglecF^–^ macrophages from *Lyz2* ^Cre/+^.*Egr2* ^fl/fl^ mice to exclude confounding effects of EGR2-sufficient alveolar macrophages) (Supplementary Figure 4). Unbiased clustering confirmed the biological replicates from each group were highly similar (Figure 3A) and differential gene expression (DEG) analysis revealed that 858 genes were differentially expressed by at least 2-fold (417 and 440 genes downregulated and upregulated, respectively) (Data File S2). Consistent with our flow cytometry analysis, *Siglec5*, which encodes SiglecF, was one of the most downregulated genes in *Egr2*-deficient alveolar macrophages (Figure 3B). Many of the most differentially expressed genes formed part of the alveolar macrophage gene set identified in our scRNA-seq analysis. Moreover, approximately 30% of the core alveolar macrophage signature identified by the ImmGen consortium (12) was altered by *Egr2* deficiency (32 genes) (Figure 3B, C), including the expression of *Spp1, Epcam, Car4* and *Fabp1*, all of which were confirmed by flow cytometry or qPCR (Figure 3D, E). The vast majority of these ‘signature’ genes was downregulated in *Egr2*-deficient macrophages compared with their *Egr2*-sufficient counterparts. Gene Ontology (GO) analysis revealed that the top pathways affected by *Egr2* deficiency were ‘Chemotaxis’, ‘Cell chemotaxis’ and ‘Immune system process’ (Supplementary Table 1). Consistent with this, the expression of chemokine receptors, such as *Ccr2* and *Cx3cr1*, was elevated in alveolar macrophages from *Lyz2* ^Cre/+^.*Egr2* ^fl/fl^ mice compared with their *Egr2* ^fl/fl^ counterparts (Figure 3F). Genes encoding antigen presentation machinery, such as *H2-Aa, H2-Eb1, Ciita* and *Cd74* were also upregulated in alveolar macrophages from *Lyz2* ^Cre/+^.*Egr2* ^fl/fl^ mice. In parallel, there was significantly greater expression of MHCII at the protein level in *Egr2*-deficient alveolar macrophages (Figure 3F). Indeed, over 50 genes upregulated in *Egr2*-deficient alveolar macrophages were genes that defined interstitial macrophages in our scRNA-seq analysis, including *Cd63, Mafb, Mmp12* and *Msr1* (Figure 3D, Data File S2). Thus, EGR2 ablation renders alveolar macrophages transcriptionally more similar to their interstitial counterparts.

Further phenotypic analysis revealed reduced expression of ‘core signature’ alveolar macrophage markers TREM1 and CD11a at protein level in the context of *Egr2* deficiency (Figure 3G). EpCAM and CD11a expression have been implicated in regulating adherence to and patrolling of the lung epithelium by alveolar macrophages (26), which suggested these behaviours may be altered by *Egr2* deficiency. However, *ex vivo* analysis of live Precision-Cut Lung Slices (PCLS) showed that CD11c^+^ alveolar macrophages remained sessile in both strains, as compared with Ly6G^+^ CD11b^+^ neutrophils moving freely in sections (Data file S3 and Supplementary Figure 5A). Nevertheless, morphodynamics analysis of macrophages demonstrated increased changes in cell shape over time (as shown by the standard deviation of cell sphericity) indicating a more active behavior of *Egr2*-deficient macrophages (Data file S4 and Supplementary Figure 5B, C). In addition to this, while we found equivalent numbers of alveolar macrophages amongst tissue digests, we obtained consistently higher numbers of alveolar macrophages in the bronchoalveolar lavage (BAL) fluid of *Lyz2* ^Cre/+^.*Egr2* ^fl/fl^ mice (Figure 3H). Taken together, these data suggest the EGR2-dependent differentiation programme may control the ability of alveolar macrophages to adhere to and interact with cells of their niche in the airways.

### EGR2 controls distinct functional characteristics of alveolar macrophages

**Figure 4:**
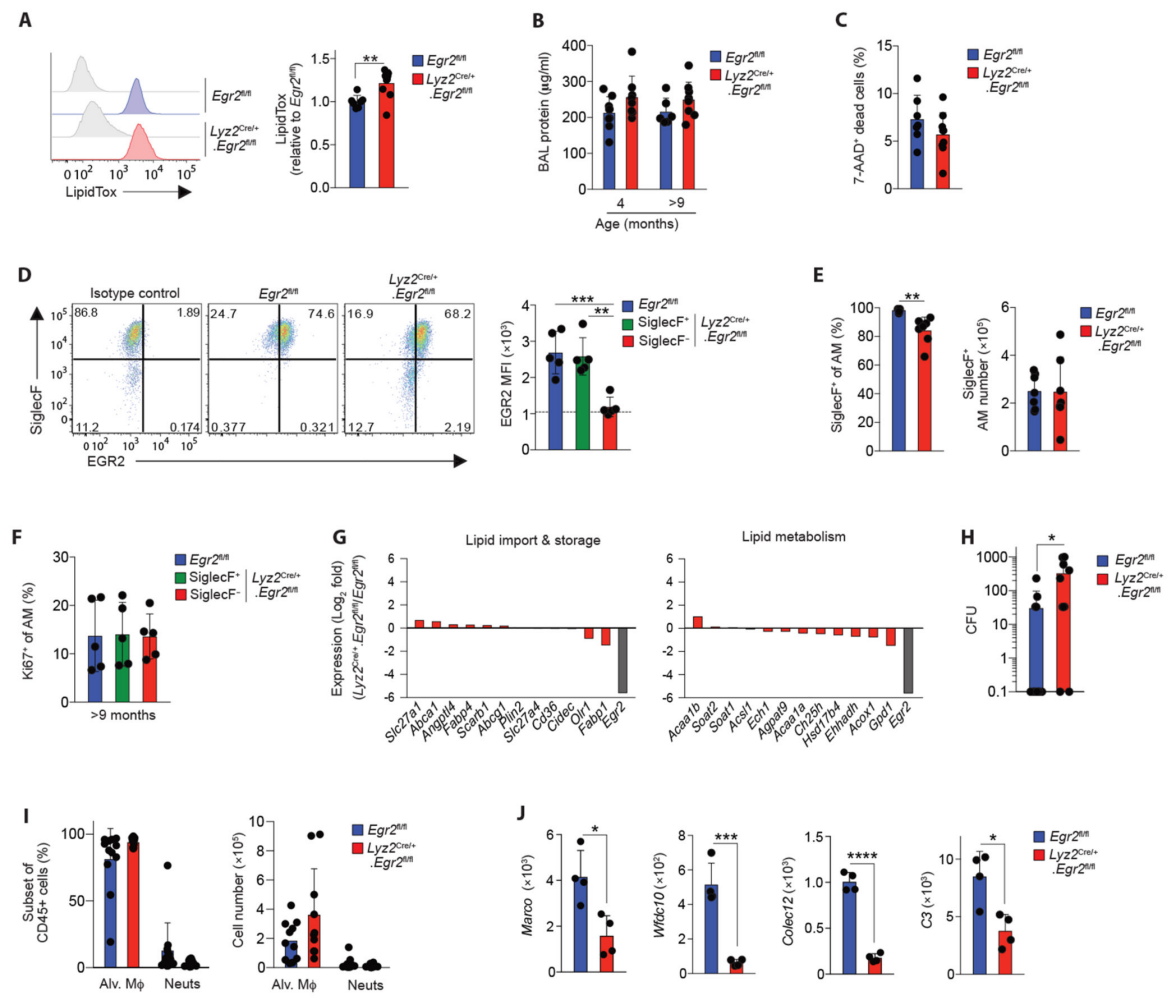
EGR2 controls distinct functional characteristics of alveolar macrophages **A.** Representative LipidTox staining of alveolar macrophages from the BAL fluid of unmanipulated adult *Egr2* ^fl/fl^ or *Lyz2* ^Cre/+^.*Egr2* ^fl/fl^ mice (*left*). Graph shows the mean fluorescence intensity (MFI) of LipidTox in alveolar macrophages from *Lyz2* ^Cre/+^.*Egr2* ^fl/fl^ mice relative to those from *Egr2* ^fl/fl^ mice. Data are from 7 (*Egr2* ^fl/fl^) and 10 (*Lyz2* ^Cre/+^.*Egr2* ^fl/fl^) mice per group pooled from three independent experiments. **p<0.01 (unpaired Student’s *t*-test). **B.** Protein levels in the BAL fluid of *Egr2* ^fl/fl^ or *Lyz2* ^Cre/+^.*Egr2* ^fl/fl^ mice at 4 or 9-12 months of age. Data are from 6-9 mice per group pooled from two independent cohorts of aged mice. **C.** Frequency of 7-AAD^+^ (dead) cells in the BAL fluid of *Egr2* ^fl/fl^ or *Lyz2* ^Cre/+^.*Egr2* ^fl/fl^ mice at 9-12 months of age. Data are from 6-9 mice per group pooled from two independent cohorts of aged mice. **D.** Representative expression of SiglecF and EGR2 by CD11c^hi^CD11b^lo^ macrophages and MFI of EGR2 by SiglecF-defined CD11c^hi^CD11b^lo^ macrophages obtained from 11-12 month old *Egr2* ^fl/fl^ or *Lyz2* ^Cre/+^.*Egr2* ^fl/fl^ mice. Data are from 5 mice per group pooled from two independent cohorts of aged mice. ** p<0.01, *** p<0.001 (One-way ANOVA followed by Tukey’s multiple comparisons post-test.) **E.** Frequency (*left*) and absolute number (*right*) of SiglecF^+^ cells amongst CD11c^hi^CD11b^lo^ macrophages obtained from 11-12 month old *Egr2* ^fl/fl^ or *Lyz2* ^Cre/+^.*Egr2* ^fl/fl^ mice. Data are from 7 mice per group pooled from three independent cohorts of aged mice. **p<0.01 (unpaired Student’s *t*-test). **F.** Frequency of Ki67^+^ cells amongst SiglecF-defined CD11c^hi^CD11b^lo^ macrophages obtained from 11-12 month old *Egr2* ^fl/fl^ or *Lyz2* ^Cre/+^.*Egr2* ^fl/fl^ mice. Data are from 5 mice per group pooled from two independent experiments. **G.** Log_2_-fold expression of genes that are implicated in lipid uptake or metabolism in alveolar macrophages as defined by (8). Expression of *Egr2* is included as a reference. **H.** Bacterial levels (colony forming units, CFU) in the BAL fluid of *Egr2* ^fl/fl^ or *Lyz2* ^Cre/+^.*Egr2* ^fl/fl^ mice 14hrs after infection. Data are from 10 (*Lyz2* ^Cre/+^.*Egr2* ^fl/fl^) or 12 (*Egr2* ^fl/fl^) mice per group pooled from three independent experiments. *p<0.05 (Mann Whitney test). **I.** Frequency (*left*) and absolute number (*right*) of CD11c^hi^CD11b^lo^ alveolar macrophages and Ly6G^+^ neutrophils in the BAL fluid of *Egr2* ^fl/fl^ or *Lyz2* ^Cre/+^.*Egr2* ^fl/fl^ mice 14hrs after infection. Data represent 10 (*Lyz2* ^Cre/+^.*Egr2* ^fl/fl^) or 11 (*Egr2* ^fl/fl^) mice per group pooled from three independent experiments. **J.** Expression of *Marco*, *Wfdc10, Colec12* and *C3* from the RNA-seq dataset (*left panels*). *p<0.05, ***p<0.001, ****p<0.0001 (unpaired Student’s *t*-test). Symbols represent individual mice in all graphs and error bars represent the standard deviation.

Individuals with mutations in *EGR2* develop peripheral neuropathies due to the crucial role for EGR2 in Schwann cell function (26). However, many of these individuals also frequently encounter respiratory complications, including recurrent pneumonias and/or restrictive pulmonary disease, and in some cases respiratory failure (26). The cause of respiratory compromise in these individuals remains unexplained. To determine if alterations in alveolar macrophage behaviour may contribute to this, we next tested the function of *Egr2*-deficient alveolar macrophages. A major homeostatic function of alveolar macrophages is the regulation of pulmonary surfactant, and the absence of alveolar macrophages results in the development of pulmonary alveolar proteinosis (14, 28–32). We first examined neutral lipid context of alveolar macrophages using LipidTox. We found a small but significant increase in the neutral lipid context in alveolar macrophages from *Lyz2* ^Cre/+^.*Egr2* ^fl/fl^ mice mice compared with *Egr2* ^fl/fl^ littermates (Figure 4A). Despite this, *Egr2* deficiency did not lead to spontaneous pulmonary alveolar proteinosis, as there were no differences in the levels of total protein in BAL fluid from *Lyz2* ^Cre/+^.*Egr2* ^fl/fl^ and *Egr2* ^fl/fl^ mice at either 4 or >9 months of age, a time at which proteinosis is detectable in *Csf2rb* ^–/–^ mice (32) (Figure 4B). Moreover, there was no detectable increase in the presence of dead cells in the BAL fluid, a common consequence of alveolar macrophage deficiency (Figure 4C). However, these results were confounded by the fact that the majority of alveolar macrophages in aged (>9 months) *Lyz2* ^Cre/+^.*Egr2* ^fl/fl^ mice was now EGR2-sufficient, with most cells expressing high levels of SiglecF (Figure 4D). These findings suggested that the cells that had escaped Cre recombination may have a competitive advantage over their EGR2-deficient counterparts. Indeed, the absolute number of SiglecF^+^ alveolar macrophages no longer differed between aged *Egr2* ^fl/fl^ and *Lyz2* ^Cre/+^.*Egr2* ^fl/fl^ mice (Figure 4E). These data are consistent with other studies noting age-related repopulation of the alveolar niche with Cre ‘escapees’ in the *Lyz2* ^Cre^ mouse (9). Notably, however, this preferential expansion of EGR2-sufficient ‘escapees’ did not relate to differences in the level of proliferation by EGR2-defined subsets, with identical frequencies of Ki67^+^ cells amongst EGR2-sufficient and -deficient macrophages in young adult and aged mice (Figure 2H & Figure 4F).

In an attempt to circumvent the confounding effects of these escapees, we generated a second strain to delete *Egr2* from macrophages by crossing *Egr2* ^fl/fl^ mice with mice expressing ‘improved’ Cre recombinase under control of the endogenous *Fgcr1* promoter (*Fcgr1* ^iCre^)(13). By using *Fcgr1* ^iCre^.*Rosa26* ^LSL-RFP^ reporter mice, we confirmed that this approach led to efficient Cre recombination in alveolar macrophages, as well as in other tissue macrophages, but not in other leukocytes (Supplementary Figure 6A). Importantly, alveolar macrophages from *Fcgr1* ^iCre/+^.*Egr2* ^fl/fl^ mice phenocopied those from *Lyz2* ^Cre/+^.*Egr2* ^fl/fl^ mice (Supplementary Figure 6B), but the frequency of Cre escapees was markedly lower in *Fcgr1* ^iCre/+^.*Egr2* ^fl/fl^ mice compared with *Lyz2* ^Cre/+^.*Egr2* ^fl/fl^ mice (Supplementary Figure 6C, D). Despite this, we did not detect the development of proteinosis or accumulation of dead cells in the BAL fluid of aged *Fcgr1* ^iCre/+^.*Egr2* ^fl/fl^ mice compared to their littermate controls (Supplementary Figure 6E). Consistent with this, *Egr2* deficiency had little if any effect on the expression of molecules associated with lipid uptake and metabolism that are characteristic of normal alveolar macrophages (8) (Figure 4G & Supplementary Figure 6F). Thus, while EGR2 is indispensable for the phenotypic identity of alveolar macrophages, it appears to be largely dispensable for lipid regulation.

We next sought to determine if EGR2-dependent differentiation controls protective immune functions of alveolar macrophages. To do so, we infected *Egr2* ^fl/fl^ mice and *Lyz2* ^Cre/+^.*Egr2* ^fl/fl^ mice with 1x10^4^ colony forming units (CFU) *Streptococcus pneumoniae*, based on previous work showing that wild type alveolar macrophages efficiently clear infection at this dose (33, 34). This showed that the majority of *Egr2* ^fl/fl^ mice (8 out of 12) had cleared infection at 14 hours post infection, whereas the majority of *Lyz2* ^Cre/+^.*Egr2* ^fl/fl^ mice (8 out of 10) had detectable bacteria in the airways at this timepoint (Figure 4H). Importantly, the failure to handle bacteria did not reflect the loss of tissue resident macrophages that can occur during inflammation or infection, as alveolar macrophages continued to dominate the airways in both groups (Figure 4I, J). Similarly, increased bacteria in *Lyz2* ^Cre/+^.*Egr2* ^fl/fl^ mice is also unlikely to reflect an effect in neutrophils, as neutrophil recruitment was negligible in both strains, and, although targeted in the *Lyz2* ^Cre^ system, neutrophils failed to express EGR2 during health or in the context of *S. pneumoniae* infection (Supplementary Figure 7A). Instead, our RNA-seq analysis showed that expression of genes encoding molecules for the recognition, opsonisation and elimination of bacteria, including *Colec12, Wfdc10, C3* and *Marco*, the latter of which has been shown to be indispensable for immunity to *S. pneumoniae* (35), were significantly reduced in *Egr2*-deficient alveolar macrophages (Figure 4K). Thus, EGR2-dependent differentiation is crucial for equipping alveolar macrophages with the machinery to capture and destroy pneumococci.

### EGR2 expression by alveolar macrophages is dependent on TGFβ and GM-CSF

**Figure 5:**
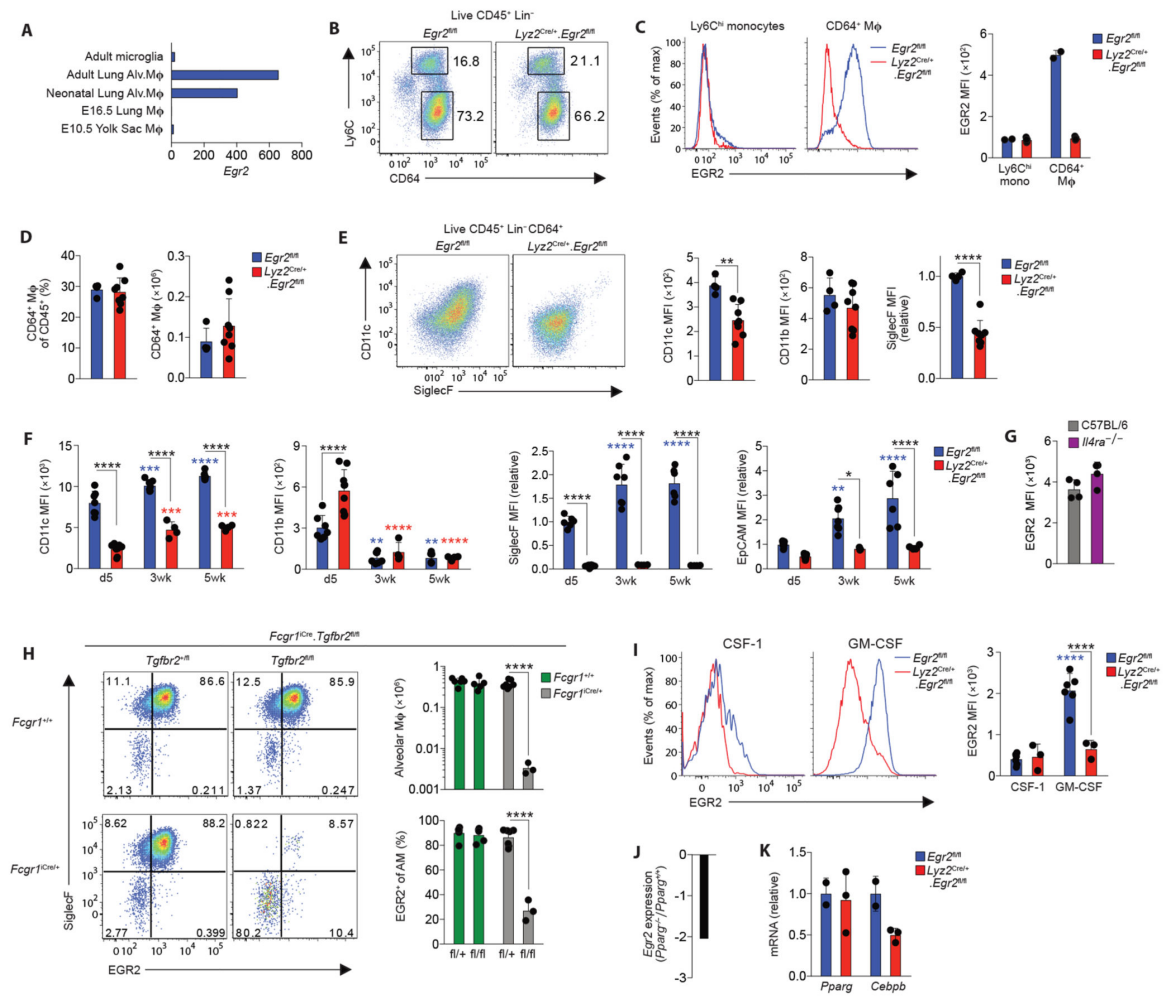
TGFβ and CSF2 drive EGR2 expression **A.** Normalised expression (by DESeq2) of *Egr2* by the indicated populations (data obtained from the ImmGen Consortium). **B.** Representative expression of Ly6C and CD64 by live CD45^+^CD3^–^CD19^–^Ly6G^–^ cells from the lungs of unmanipulated newborn *Egr2* ^fl/fl^ or *Lyz2* ^Cre/+^.*Egr2* ^fl/fl^ mice. Data are from one of two independent experiments performed. **C.** Histograms show representative expression of EGR2 by CD64^+^ ‘pre-alveolar macrophages’ and Ly6C^hi^ monocytes from the lungs of unmanipulated newborn *Egr2* ^fl/fl^ or *Lyz2* ^Cre/+^.*Egr2* ^fl/fl^ mice and bar chart shows the mean fluorescent intensity (MFI) of EGR2 expression by these cells. Data are from one of two independent experiments performed with 2 (*Egr2* ^fl/fl^) or 5 (*Lyz2* ^Cre/+^.*Egr2* ^fl/fl^) mice per group. **D.** Frequency and absolute number of CD64^+^ ‘pre-alveolar macrophages’ from mice in **B.** Data are pooled from two independent experiments with 4 (*Egr2* ^fl/fl^) or 8 (*Lyz2* ^Cre/+^.*Egr2* ^fl/fl^) mice per group. **E.** FACS plots show representative expression of CD11c and SiglecF by CD64^+^ ‘pre-alveolar macrophages’ from mice in **B** and bar chart shows the MFI of CD11c, SiglecF and CD11b expression by these cells. Data are pooled from two independent experiments with 4 (*Egr2* ^fl/fl^) or 8 (*Lyz2* ^Cre/+^.*Egr2* ^fl/fl^) mice per group. **F.** MFI of CD11c and CD11b and relative MFI of SiglecF and EpCAM (relative to cells from d5 old *Egr2* ^fl/fl^ mice) expression by CD11c^hi^CD11b^lo^ alveolar macrophages obtained from unmanipulated *Egr2* ^fl/fl^ or *Lyz2* ^Cre/+^.*Egr2* ^fl/fl^ mice at the indicated ages. Data are pooled from two independent experiments with 4-9 mice per group. Coloured * denote significance between d5 and 3 and 5 weeks within the *Egr2* ^fl/fl^ (blue) and *Lyz2* ^Cre/+^.*Egr2* ^fl/fl^ (red) data. **p<0.01, ***p<0.001, ****p<0.0001 (Two-way ANOVA with Tukey’s multiple comparisons test). **G.** Representative expression of EGR2 by alveolar macrophages from adult WT (C57BL/6) and *Il4ra* ^–/–^ adult mice. Data from one experiment with 4 mice per group. **H.** Representative expression of EGR2 and SiglecF by CD11c^hi^CD11b^lo^ alveolar macrophages obtained from lungs of neonatal (d8) *Fcgr1* ^iCre/+^.*Tgfbr2* ^fl/fl^ and littermate controls. Bar charts show the absolute numbers of CD11c^hi^CD11b^lo^ alveolar macrophages (*upper*) and the mean frequency of EGR2^+^ cells amongst CD11c^hi^CD11b^lo^ alveolar macrophages (*lower*). Data are pooled from two independent experiments with 3-7 mice per group. **** p<0.0001 (One-way ANOVA followed by Tukey’s multiple comparisons post-test). **I.** Representative expression of EGR2 (*left*) and MFI of EGR2 (*right*) by FACS-purified Ly6C^hi^ monocytes cultured *in vitro* with recombinant CSF-1 (20ng/ml) or GM-CSF (20ng/ml) for five days. Symbols represent monocytes isolated from individual mice. Data are from 6 *Egr2* ^fl/fl^ (Cre^–^) or 3 *Lyz2* ^Cre^.*Egr2* ^fl/fl^ (Cre^+^) mice per group pooled from two independents experiment. **** p<0.0001 (Two-way ANOVA followed by Tukey’s multiple comparisons post-test). Coloured * denote significance between CSF-1 and GM-CSF within the *Egr2* ^fl/fl^ (blue) and *Lyz2* ^Cre^.*Egr2* ^fl/fl^ (red) data. **J.** Relative expression of *Egr2* by alveolar macrophages obtained from *Pparg* ^fl/fl^ or *Itgax* ^Cre^.*Pparg* ^fl/fl^ mice from the ImmGen Consortium. **K.** qPCR analysis of *Pparg* and *Cebpb* mRNA by BAL cells from unmanipulated adult *Egr2* ^fl/fl^ or *Lyz2* ^Cre^.*Egr2* ^fl/fl^ mice. Data represent 2 *Egr2* ^fl/fl^ or 4 *Lyz2* ^Cre^.*Egr2* ^fl/fl^ mice per group. Symbols represent individual mice in all graphs and error bars represent the standard deviation.

Alveolar macrophages derive from foetal monocytes that seed the developing lung in the late gestational period (7). To determine the point at which EGR2 is first expressed, we assessed EGR2 expression by E10.5 yolk sac macrophages, by macrophages in the embryonic lung (E16.5) and by CD11c^hi^CD11b^lo^ alveolar macrophages in the neonatal and adult lung using the ImmGen database. This revealed that *Egr2* was absent from yolk sac macrophages and macrophages in the embryonic lung at E16.5, but it was expressed by both neonatal and adult alveolar macrophages (Figure 5A), suggesting that it is induced during alveolarization in the neonatal period. Consistent with this, we found high expression of EGR2 at protein level by neonatal (d1) CD64^+^ lung macrophages (sometimes referred to as ‘pre-alveolar macrophages’) in *Egr2* ^fl/fl^ mice; as expected, this expression was deleted efficiently in *Lyz2* ^Cre/+^.*Egr2* ^fl/fl^ mice (Figure 5B, C). Importantly, Ly6C^hi^ monocytes in the lung of d1 neonatal mice lacked any expression of EGR2 (Figure 5B, C), reinforcing the selectivity of EGR2 expression even at this highly dynamic stage of myeloid cell development in the lung. Consistent with our analysis of mature alveolar macrophages in adult mice, *Egr2* deletion had no impact on the frequency and absolute number of pre-alveolar macrophages (Figure 5D). However, phenotypic differences were already apparent in macrophages from *Lyz2* ^Cre/+^.*Egr2* ^fl/fl^ mice at this stage, with reduced CD11c and SiglecF expression which persisted into adulthood (Figure 5E, F). In parallel, EpCAM expression was absent from alveolar macrophages in the neonatal period and was progressively upregulated with age in an EGR2-dependent manner (Figure 5F). CD11b expression, which is downregulated in mature alveolar macrophages, was found on pre-alveolar macrophages in both *Egr2* ^fl/fl^ and *Lyz2* ^Cre/+^.*Egr2* ^fl/fl^ mice, and it was downregulated to the same extent with age in both strains.

We next set out to determine the environmental factors that drive EGR2 expression. Many studies employing *in vitro* culture systems have described EGR2 expression as a feature of ‘alternatively activated’ macrophages, dependent on IL-4R signalling (36, 37). Importantly, expression of EGR2 by alveolar macrophages was independent of IL-4R signalling (Figure 5G & Supplementary Figure 6G), as were key EGR2-dependent phenotypic traits, such as SiglecF and EpCAM expression (Supplementary Figure 6F). TGF-β has recently been shown to be crucial for the development of alveolar macrophages (9) and thus we next explored if the TGF-β-TGF-βR axis drives expression of EGR2. To do so, we generated a new mouse line by crossing *Fcgr1* ^iCre^ mice to mice with LoxP sites flanking the *Tgfbr2* allele (*Tgfbr2* ^fl/fl^). Consistent with the crucial role for TGF-βR in controlling alveolar macrophage development (9), there was a paucity of alveolar macrophages in the lungs of neonatal *Fcgr1* ^iCre/+^.*Tgfbr2* ^fl/fl^ compared with *Fcgr1* ^+/+^.*Tgfbr2* ^fl/fl^ and *Fcgr1* ^iCre/+^.*Tgfbr2* ^fl/+^ controls (Figure 5H). Strikingly, while CD11c^+^CD11b^lo^ alveolar macrophages expressed high levels of EGR2 in control groups, EGR2 expression was largely abolished in *Fcgr1* ^iCre/+^.*Tgfbr2* ^fl/fl^ mice, demonstrating that TGF-βR signalling is vital for EGR2 induction *in vivo*. As *Fcgr1* ^iCre/+^.*Tgfbr2* ^fl/fl^ developed fatal seizures between d14 and d21 of age, perhaps reflecting the indispensable role for TGF-βR in controlling microglia activity (38, 39), we were unable to carry out further analyses using this strain.

Given the central role for GM-CSF in alveolar macrophage development, we also assessed the role of GM-CSF in driving EGR2 expression using an *in vitro* culture system in which Ly6C^hi^ monocytes from bone marrow were FACS-purified and cultured with recombinant CSF-1 or GM-CSF. This revealed that GM-CSF was also capable of driving EGR2 expression in this system (Figure 5I). Given that GM-CSF receptor and TGF-βR signalling is known to induce expression of PPAR-γ (8, 9, 14), we next determined if PPAR-γ is upstream of EGR2. Analysis of a publicly available dataset (ImmGen) comparing the transcriptional profile of *Pparg*-sufficient and -deficient alveolar macrophages revealed downregulation (2.1-fold change) of *Egr2* in the context of *Pparg* deficiency (Figure 5j). In contrast, *Pparg* expression was unaffected in alveolar macrophages from *Lyz2* ^Cre/+^.*Egr2* ^fl/fl^ mice (Figure 5K), suggesting EGR2 is downstream of PPAR-γ. Another transcription factor implicated in controlling alveolar macrophage differentiation is C/EBPβ (15) and EGR2 has been shown to modulate C/EBPβ *in vitro* (36). *Egr2* deficiency led to reduced expression of C/β at mRNA (Figure 5K) and protein level (Supplementary Figure 6H). Taken together, these data support the premise that EGR2 expression by alveolar macrophages is induced by TGF-β and GM-CSF in a PPAR-γ-dependent manner in the neonatal period and this in turn induces expression of C/EBPβ to drive tissue-specific differentiation.

### *Egr2* deficiency confers a competitive disadvantage on alveolar macrophages

**Figure 6:**
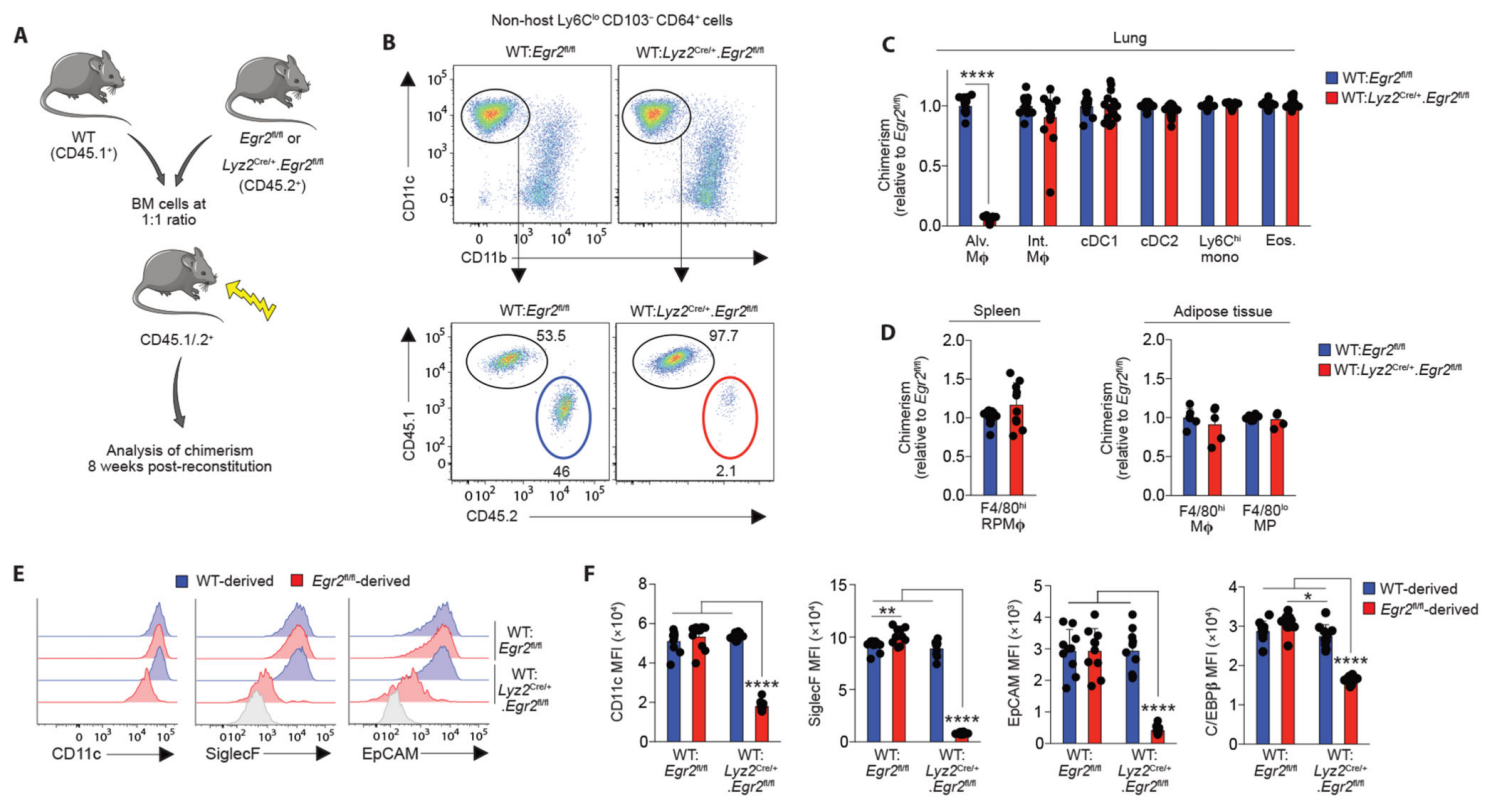
*Egr2* deficiency confers a competitive disadvantage on alveolar macrophages **A.** Schematic of the generation of mixed bone marrow chimeric mice **B.** Representative expression of CD11c and CD11b by Ly6C^lo^CD64^+^ macrophages amongst live CD45^+^CD3^–^CD19^–^Ly6G^–^CD103^–^ cells (*upper panels*) and representative expression of CD45.1 and CD45.2 by CD11c^hi^CD11b^lo^ alveolar macrophages (*lower panels*) from WT:*Egr2* ^fl/fl^ or WT:*Lyz2* ^Cre/+^.*Egr2* ^fl/fl^ chimeric mice. **C.** Contribution of *Egr2* ^fl/fl^ BM to the indicated lung myeloid populations in WT:*Lyz2* ^Cre/+^.*Egr2* ^fl/fl^ chimeric mice relative to WT:*Egr2* ^fl/fl^ mice. Chimerism was normalised to Ly6C^hi^ blood monocytes before normalisation of *Lyz2* ^Cre/+^.*Egr2* ^fl/fl^ to *Egr2* ^fl/fl^. Data are from 15 (WT:*Lyz2* ^Cre/+^.*Egr2* ^fl/fl^) or 16 (WT:*Egr2* ^fl/fl^) mice per group pooled from three independent experiments. **** p<0.0001 (Student’s *t*-test with Holm-Sidak correction). **D.** Contribution of *Egr2* ^fl/fl^ BM to splenic red pulp F4/80^hi^ macrophages and F4/80-defined mononuclear phagocytes in adipose tissue from chimeric in **C.** Spleen data are from 15 (WT:*Lyz2* ^Cre/+^.*Egr2* ^fl/fl^) or 16 (WT:*Egr2* ^fl/fl^) mice per group pooled from three independent experiments and adipose data from 5 (WT:*Lyz2* ^Cre/+^.*Egr2* ^fl/fl^) or 6 (WT:*Egr2* ^fl/fl^) mice per group pooled from two independent experiments. **E.** Representative expression of CD11c, SiglecF and EpCAM by WT- and *Egr2* ^fl/fl^-derived alveolar macrophages in WT:*Egr2* ^fl/fl^ or WT:*Lyz2* ^Cre/+^.*Egr2* ^fl/fl^ chimeric mice. Shaded histograms represent FMO controls. **F.** Mean fluorescent intensity (MFI) of CD11c, SiglecF, EpCAM and C/EBPβ expression by WT- and *Egr2* ^fl/fl^-derived alveolar macrophages in WT:*Egr2* ^fl/fl^ or WT:*Lyz2* ^Cre/+^.*Egr2* ^fl/fl^ chimeric mice. Data represent 10 mice per group from one experiment of three performed. **** p<0.0001 (One-way ANOVA followed by Tukey’s multiple comparisons post-test). Symbols represent individual mice in all graphs and error bars represent the standard deviation.

Given the observation that EGR2-sufficient alveolar macrophages come to dominate the airspace of *Lyz2* ^Cre/+^.*Egr2* ^fl/fl^ mice, we next set out to determine if EGR2 deletion confers an intrinsic competitive disadvantage on alveolar macrophages. To this end, we generated mixed bone marrow chimeric mice by reconstituting lethally irradiated WT (CD45.1^+^/.2^+^) mice with a 1:1 ratio of WT (CD45.1^+^) and either *Egr2* ^fl/fl^ or *Lyz2* ^Cre/+^.*Egr2* ^fl/fl^ (CD45.2^+^) bone marrow cells (Figure 6A). 8 weeks after reconstitution, we found that *Egr2*-deficient and *Egr2* sufficient bone marrow contributed equally to the pools of monocytes, interstitial macrophages and dendritic cell subsets in the lung (Figure 6B, C). In contrast, alveolar macrophages were derived almost exclusively from WT BM in WT:*Lyz2* ^Cre/+^.*Egr2* ^fl/fl^ chimeric mice, whereas they were derived equally from both BM sources in WT:*Egr2* ^fl/fl^ chimeric mice (Figure 6B, C). These effects were not a general feature of macrophages derived from *Egr2*-deficient bone marrow, as *Egr2* deletion did not adversely affect the replenishment of splenic red pulp or adipose tissue macrophages (Figure 6D). The mixed BM chimeric model also confirmed that the phenotypic differences seen in alveolar macrophages from intact *Lyz2* ^Cre/+^.*Egr2* ^fl/fl^ mice were due to cell intrinsic loss of EGR2, rather than effects of *Egr2* deficiency on the lung environment (Figure 6E, F). We also used this system to confirm the reduced expression of C/EBPβ by alveolar macrophages deriving from *Lyz2* ^Cre/+^.*Egr2* ^fl/fl^ bone marrow (Figure 6F).Taken together, these results demonstrate that cell intrinsic EGR2 is indispensable for the differentiation of alveolar macrophages and repopulation of the alveolar niche following radiation-induced depletion.

### Bone marrow-derived monocytes replenish the alveolar macrophage niche following lung injury

**Figure 7:**
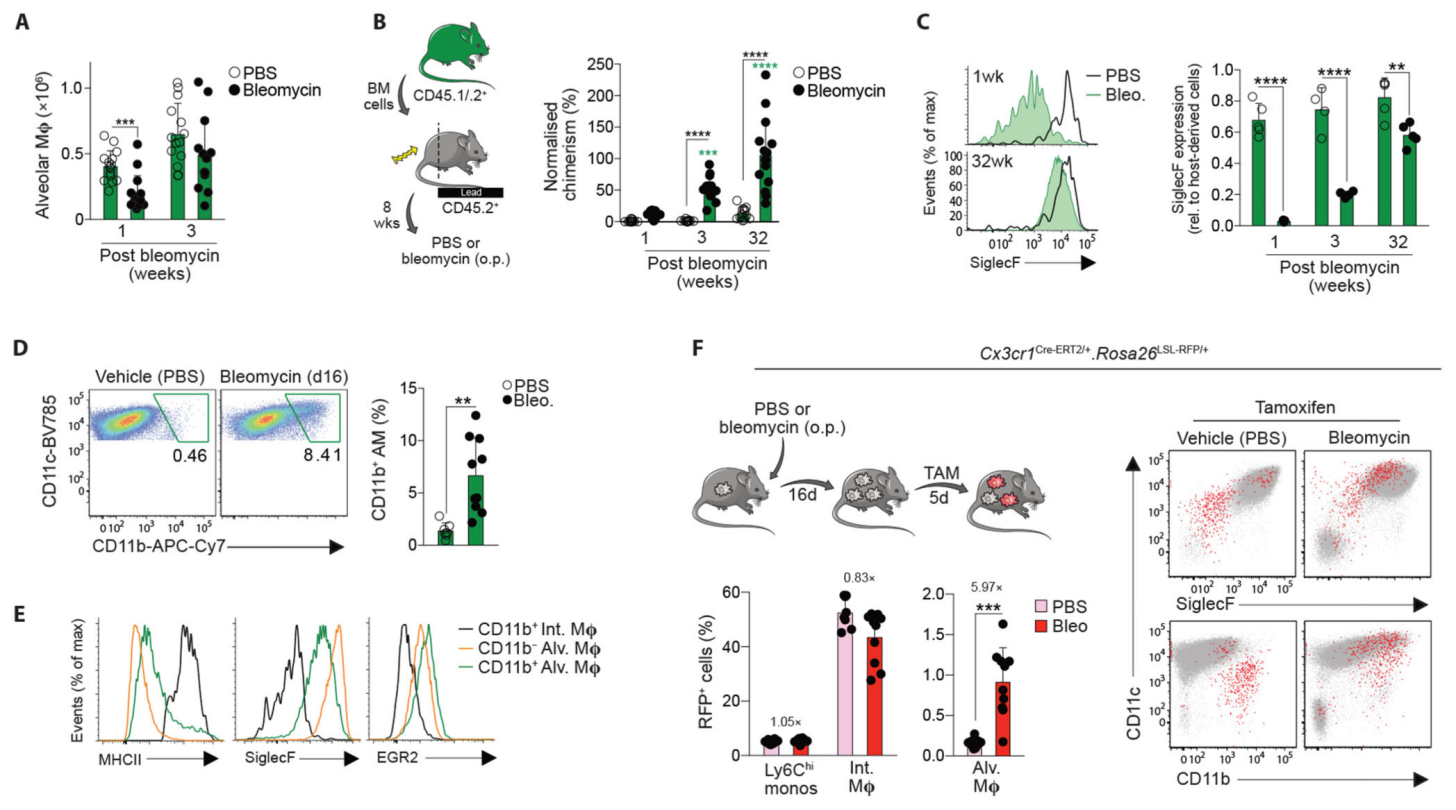
Monocyte-derived, CX3CR1^+^ parenchymal macrophages can replenish the alveolar macrophage niche following injury **A.** Absolute numbers of alveolar macrophages 1- and 3-weeks following bleomycin administration or PBS vehicle control. Data are pooled from two independent experiments at each time point with 13-15 mice per group. ***p<0.001 (unpaired Student’s *t* test with Holm-Sidak correction). **B.** Non-host chimerism of alveolar macrophages in tissue protected bone marrow chimeric mice at 1-, 3- or 32-weeks following administration of bleomycin or PBS vehicle control. Chimerism is normalised to Ly6C^hi^ blood monocytes. Data are pooled from two independent experiments at each time point with 13-15 mice per group. ***p<0.001, ****p<0.0001 (Two-way ANOVA with Tukey’s multiple comparisons test) **C.** Expression of SiglecF by CD11c^hi^CD11b^lo^ alveolar macrophages from the lung of mice in **B**. at 1 week and 32 weeks post bleomycin or PBS administration. Data are from one of two independent experiments at each time point with 4 mice per group. ***p<0.001, ****p<0.0001 (Two-way ANOVA with Tukey’s multiple comparisons test). **D.** Representative expression of CD11c and CD11b by CD11c^hi^CD64^+^ cells obtained by BAL from WT mice two weeks after instillation of bleomycin or vehicle control (*left)*. Graph shows the mean frequency of CD11b^+^ alveolar macrophages (*right*). Data are pooled from two independent experiments with 7 (PBS) or 10 (bleomycin) mice per group. **p<0.01 (unpaired Student’s *t* test). **E**. Representative expression of MHCII, SiglecF and EGR2 by CD11b^+^ interstitial macrophages and CD11b-defined CD11c^hi^ alveolar macrophages. **F.** Experimental scheme for the induction of lung injury and tamoxifen administration in *Cx3cr1* ^Cre-ERT2/+^.*Rosa26* ^LSL-RFP/+^ fate mapping mice. Lower graphs show the levels of recombination in Ly6C^hi^ monocytes, CD64^+^ interstitial macrophages and alveolar macrophages from *Cx3cr1* ^Cre-ERT2/+^.*Rosa26* ^LSL-RFP/+^ mice administered bleomycin or vehicle control. Representative expression of CD11c, SiglecF and CD11b by RFP^+^ (red) or RFP^–^ (grey) cells present in the BAL fluid of *Cx3cr1* ^Cre-ERT2/+^.*Rosa26* ^LSL-RFP/+^ mice 3 weeks after bleomycin or vehicle instillation. Graphs show the mean fluorescent intensity (MFI) of CD11c and SiglecF expression by RFP^+^ cells. Symbols represent individual mice in all graphs and error bars represent the standard deviation.

Loss of tissue resident macrophages is a frequent consequence of inflammation, including in the lung (40). Thus, given that *Egr2*-deficient macrophages failed to replenish the alveolar niche following radiation treatment, we next sought to determine if EGR2 plays a role in macrophage repopulation following lung injury. The chemotherapeutic agent bleomycin is a common model of chronic lung injury and self-resolving pulmonary fibrosis (41), which is characterised by initial loss of alveolar macrophages during the inflammatory phase (day 7), followed by repopulation during the fibrotic and resolution phases (from day 14 onwards) (Figure 7A). To determine if bone marrow-derived monocytes contribute to the alveolar macrophage compartment following bleomycin-induced injury, we used tissue protected bone marrow chimeric mice to assess replenishment kinetics without exposing the lung to the additional insult of ionising radiation (Figure 7B). Consistent with previous studies (42), we found that bleomycin instillation led to progressive replacement of resident alveolar macrophages by BM-derived cells, with the entire alveolar macrophage compartment being replaced at 32 weeks post injury (Figure 7B). Interestingly, recently arrived, monocyte-derived alveolar macrophages expressed low-intermediate levels of SiglecF, with acquisition of SiglecF requiring long-term residence in the airway (Figure 7C).

We next interrogated this process further to determine if monocyte-derived macrophages that accumulate in the lung parenchyma during injury can subsequently mature into alveolar macrophages during tissue repair (42, 43). Indeed, during the recovery phase of disease, we noted the presence of cells with features of both alveolar and interstitial macrophages (CD11c^hi^CD11b^+^ MHCII^+^CD64^hi^) in the BAL fluid (Figure 7D), and these cells expressed intermediate levels of SiglecF (Figure 7E), indicative of recent monocyte origin. To examine the relationship of these ‘hybrid’ cells found in the airways to elicited monocyte-derived macrophages in the lung parenchyma more directly, we performed fate mapping studies using *Cx3cr1* ^Cre-ERT2/+^.*Rosa26* ^LSL-RFP/+^ reporter mice, in which administration of tamoxifen leads to irreversible expression of RFP by CX3CR1 expressing cells (44, 45) (Figure 7F). Administration of tamoxifen led to labelling of 40-50% of CD11b^+^ parenchymal macrophages in both healthy lung and at d21 post bleomycin administration (Figure 7F). No recombination was seen in *Cx3cr1* ^Cre-ERT2/+^.*Rosa26* ^LSL-RFP/+^ mice in the absence of tamoxifen (Supplementary Figure 8A). Although very low levels of recombination were detected in control alveolar macrophages, a clear population of RFP^+^ cells could be detected in the BAL of the recipients of bleomycin following tamoxifen treatment (Figure 7F). As monocytes are poorly labelled in this system and *Cx3cr1* levels do not change in *bona fide* resident alveolar macrophages in response to bleomycin treatment (Supplementary Figure 8B), these RFP^+^ cells likely represent fate-mapped, monocyte-derived CX3CR1^+^ cells. In line with this, RFP^+^ cells had a ‘hybrid’ CD11c^hi^CD11b^+^SiglecF^int^ profile, supporting the idea that these represent transitional cells (Figure 7F). Thus, following bleomycin-induced injury, the alveolar macrophage compartment is restored, in part, by monocytes that transition through a CX3CR1^hi^ state.

### EGR2 is indispensable for alveolar macrophage repopulation and tissue repair following lung injury

**Figure 8:**
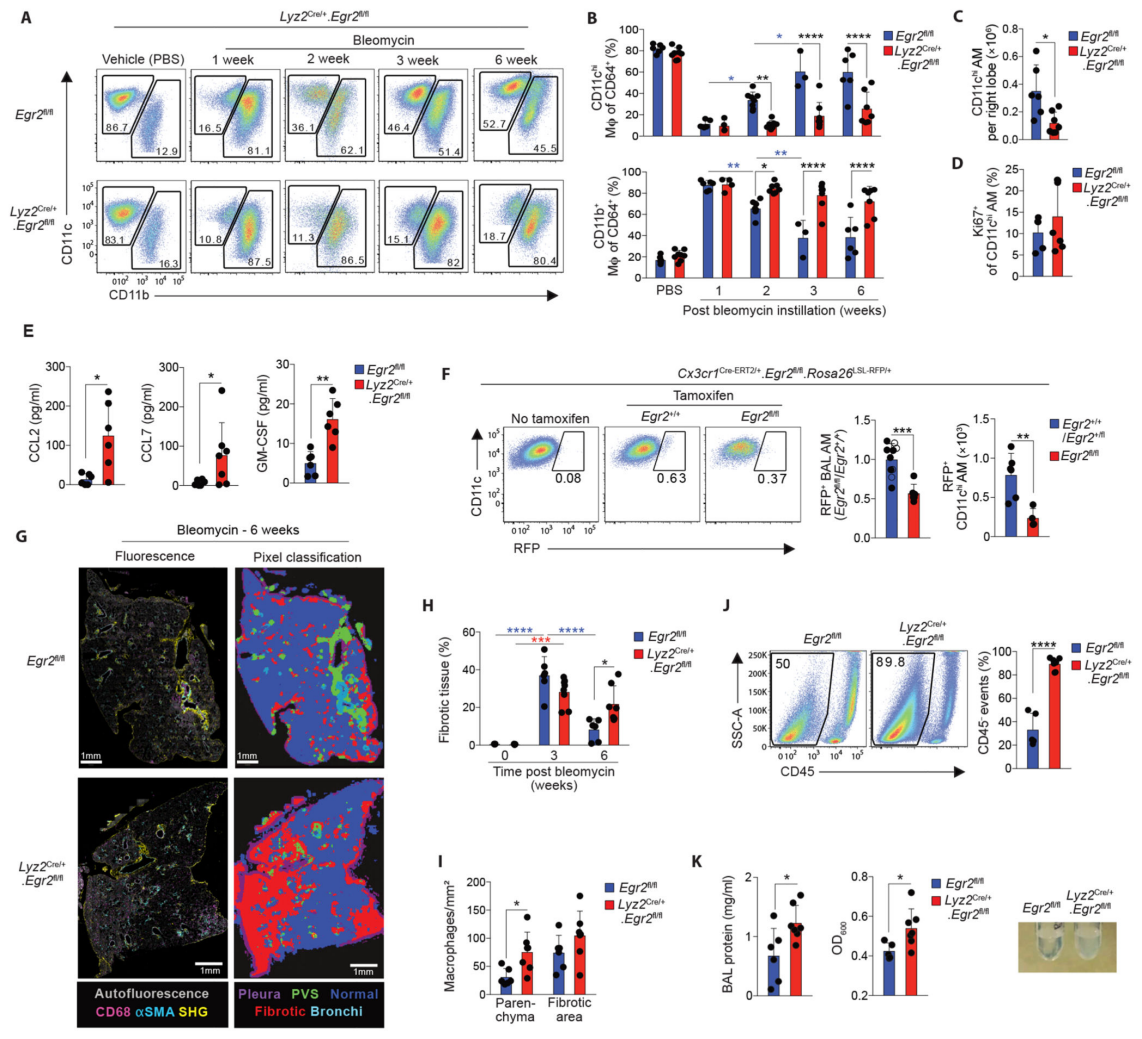
EGR2 is indispensable for the repopulation of the alveolar macrophage niche and tissue repair following lung injury **A**. Representative expression of CD11c and CD11b by live CD45^+^CD3^–^CD19^–^Ly6G^–^CD64^+^ cells from the lungs of *Egr2* ^fl/fl^ and *Lyz2* ^Cre/+^.*Egr2* ^fl/fl^ mice at 1, 2, 3 or 6 weeks post bleomycin or vehicle controls. **B.** Frequency of CD11c^hi^CD11b^lo^ alveolar macrophages and CD11c^var^CD11b^+^ cells from mice in **A.** Data are pooled from at least two independent experiments at each time point with 3-7 mice per group. *p<0.05. **p<0.01, p<0.001, ****p<0.0001 (Two-way ANOVA with Tukey’s multiple comparisons test). **C.** Absolute number of CD11c^hi^CD11b^lo^ alveolar macrophages in lungs six weeks post bleomycin instillation. Data are pooled from two independent experiments with 6 (*Egr2* ^fl/fl^) or 7 (*Lyz2* ^Cre/+^.*Egr2* ^fl/fl^) mice per group. *p<0.05 (Mann Whitney test). **D.** Frequency of Ki67^+^ CD11c^hi^CD11b^lo^ alveolar macrophages in lungs six weeks post bleomycin instillation. Symbols represent individual mice. Data are pooled from two independent experiments with 6 (*Egr2* ^fl/fl^) or 7 (*Lyz2* ^Cre/+^.*Egr2* ^fl/fl^) mice per group. **E.** CCL2, CCL7 and GM-CSF levels in BAL fluid obtained from *Egr2* ^fl/fl^ and *Lyz2* ^Cre/+^.*Egr2* ^fl/fl^ mice six weeks post bleomycin instillation. Data are pooled from two independent experiments with 6 mice per group. *p<0.05, Mann Whitney test (CCL2, CCL7), **p<0.01 (unpaired Student’s *t* test; GM-CSF). **F.** Representative expression of RFP by CD11c^hi^CD64^+^ alveolar macrophages present in the BAL fluid of *Cx3cr1* ^Cre-ERT2/+^.*Rosa26* ^LSL-RFP/+^.*Egr2* ^fl/fl^ and their *Cx3cr1* ^Cre-ERT2/+^.*Rosa26* ^LSL-RFP/+^.*Egr2* ^+/+^ (open circles) or *Cx3cr1* ^Cre-ERT2/+^.*Rosa26* ^LSL-RFP/+^.*Egr2* ^fl/+^ (solid circles) controls 3 weeks following instillation of bleomycin or vehicle control. Graphs show the relative frequency (*left*) or absolute number (*right*) of RFP^+^ alveolar macrophages present in the BAL fluid. Data are from one experiment of two (number) with 6 (*Egr2* ^+/+^ [open symbols]/*Egr2* ^fl/+^ [filled symbols]) or 4 mice (*Egr2* ^fl/fl^) per group, or pooled from two independent experiments (frequency) at each time point with 10 (*Egr2* ^+/+^ [open symbols]/*Egr2* ^fl/+^ [filled symbols]) or 6 (*Egr2* ^fl/fl^) per group. ** p<0.01, ***p<0.001 (Unpaired Student’s t test). **G.** 2-photon fluorescence imaging of lung tissue from adult *Egr2* ^fl/fl^ and *Lyz2* ^Cre/+^.*Egr2* ^fl/fl^ mice 6 weeks following bleomycin administration. Sections were stained with CD68, aSMA and DAPI. Autofluorescence is depicted in grey and collagen was detected by second harmonic generation (SHG). Pixel classification was used to segment lung regions of interest: (1) normal lung parenchyma/alveolar tissue, (2) pathologic/fibrotic tissue and (3) collagen rich areas (perivascular/bronchial spaces and pleura) were segmented to avoid false fibrotic region detection. **H.** Quantification of fibrotic score of lung tissue from *Egr2* ^fl/fl^ and *Lyz2* ^Cre/+^.*Egr2* ^fl/fl^ 3 or 6 weeks following bleomycin administration or PBS controls (from 3 week time point). See Fig. S10. Data are pooled from two independent experiments with 6 (*Egr2* ^fl/fl^) or 7 (*Lyz2* ^Cre/+^.*Egr2* ^fl/fl^) mice per group. *p<0.05, **p<0.01, ***p<0.001, ****p<0.0001 (Two-way ANOVA followed by Tukey’s multiple comparisons test). **I.** Quantification of macrophage density in the parenchyma and fibrotic areas of lung tissue from *Egr2* ^fl/fl^ and *Lyz2* ^Cre/+^.*Egr2* ^fl/fl^ 6 weeks following bleomycin administration. See Fig. S10. Data are pooled from two independent experiments with 6 (*Egr2* ^fl/fl^) or 7 (*Lyz2* ^Cre/+^.*Egr2* ^fl/fl^) mice per group. *p<0.05 (Student’s t test with Holm-Sidak correction for multiple tests). **J.** SSC-A profile and expression of CD45 by BAL obtained from *Egr2* ^fl/fl^ and *Lyz2* ^Cre/+^.*Egr2* ^fl/fl^ 6 weeks following bleomycin administration. Graph shows the mean frequency of CD45^+^ cells amongst all live, single events. Symbols represent individual mice. Data are pooled from two independent experiments with 5 (*Egr2* ^fl/fl^) or 7 (*Lyz2* ^Cre/+^.*Egr2* ^fl/fl^) mice per group. ****p<0.0001 (unpaired Student’s t test). **K.** Total protein concentration (*left*), turbidity (*centre*) and representative pictures (*right*) of BAL fluid from *Egr2* ^fl/fl^ and *Lyz2* ^Cre/+^.*Egr2* ^fl/fl^ 6 weeks following bleomycin administration. Symbols represent individual mice. Data are pooled from two independent experiments with 6 (*Egr2* ^fl/fl^) or 7 (*Lyz2* ^Cre/+^.*Egr2* ^fl/fl^) mice per group. *p<0.05 (Mann Whitney test). Symbols represent individual mice in all graphs and error bars represent the standard deviation.

Given that transitional CD11b^+^SiglecF^int^ cells also expressed EGR2, contrasting with its restriction to SiglecF^hi^ alveolar macrophages in health (Figure 7E), we examined whether EGR2 is necessary for the replenishment of the alveolar niche during recovery from bleomycin-induced injury. To do this, we administered bleomycin to *Lyz2* ^Cre/+^.*Egr2* ^fl/fl^ mice and their *Egr2* ^fl/fl^ littermates and assessed macrophage dynamics in total lung digests. The inflammatory phase of this disorder (day 7) was associated with accumulation of CD11b^+^ macrophages and this occurred to the same extent in both strains (Figure 8A, B). Consistent with recent reports (46), the CD11b^+^CD64^+^ interstitial macrophage population was heterogeneous during the fibrotic phase of disease (d14-d21), with MHCII^+^ and MHCII^lo^CD36^+^Lyve1^+^ subsets. This pattern was identical in between *Egr2* ^fl/fl^ and *Lyz2* ^Cre/+^.*Egr2* ^fl/fl^ groups, as were the numbers of Ly6C^hi^ monocytes and neutrophils (Supplementary Figure 9A-C). We did however detect a significant reduction in eosinophils in the lung of *Lyz2* ^Cre/+^.*Egr2* ^fl/fl^ mice compared with *Egr2* ^fl/fl^ littermates, despite eosinophils lacking EGR2 expression and no differences in the level of eosinophil chemoattractant CCL11 (Supplementary Figure 9D-F).

A reduction in alveolar macrophages was observed in both groups on day 7 after administration of bleomycin. Although this began to be restored by day 14 in *Egr2* ^fl/fl^ control mice, this did not occur in *Lyz2* ^Cre/+^.*Egr2* ^fl/fl^ mice and indeed, the alveolar macrophage compartment remained significantly reduced in *Lyz2* ^Cre/+^.*Egr2* ^fl/fl^ mice compared with *Egr2* ^fl/fl^ littermates even after 6 weeks (Figure 8A-C), suggesting EGR2 is indispensable for the repopulation of the alveolar macrophage niche following bleomycin-induced injury. The lack of repopulation in *Lyz2* ^Cre/+^.*Egr2* ^fl/fl^ mice did not appear to reflect an inability of *Egr2*-deficient macrophages to proliferate, as the proportion of Ki67^+^ proliferating cells was equivalent across both strains (Figure 8D). Equally, this also did not reflect a lack of chemoattractants in the airways to recruit monocyte-derived cells, as both CCL2 and CCL7 were actually elevated in *Lyz2* ^Cre/+^.*Egr2* ^fl/fl^ mice compared with control littermates (Figure 8E). Similarly, GM-CSF levels were elevated in the BAL fluid of *Lyz2* ^Cre/+^.*Egr2* ^fl/fl^ mice, ruling out the possibility that lack of appropriate growth factors is responsible for defective alveolar macrophage differentiation in the absence of EGR2 (Figure 8E). Instead, these data suggested that *Egr2* deficiency led to an intrinsic inability of bone marrow-derived cells to repopulate the macrophage niche. To test this directly, we crossed *Cx3cr1* ^Cre-ERT2/+^.*Rosa26* ^LSL-RFP/+^ mice with *Egr2* ^fl/fl^ mice to allow for temporal RFP labelling of CX3CR1-expressing cells and *Egr2* deficiency in the same animal. We administered tamoxifen during the period of alveolar macrophage reconstitution (d16 to d21) and assessed the presence of RFP-labelled cells amongst alveolar macrophages. Although labelling efficiencies were low, most likely reflecting the short period of tamoxifen induction and the dynamic nature of macrophage repopulation, compared with tamoxifen-treated controls (*Cx3cr1* ^Cre-ERT2/+^.*Rosa26* ^LSL-RFP/+^.*Egr2* ^+/+^ or *Cx3cr1* ^Cre-ERT2/+^.*Rosa26* ^LSL-RFP/+^.*Egr2* ^fl/+^ mice), we found a marked reduction in the frequency of RFP^+^ alveolar macrophages in the BAL of tamoxifen treated *Cx3cr1* ^Cre-ERT2/+^.*Rosa26* ^LSL-RFP/+^.*Egr2* ^fl/fl^ mice during lung repair (Figure 8F), demonstrating that EGR2 controls the post-injury repopulation of the alveolar macrophage compartment by CX3CR1^+^ cells.

To determine the consequence of the failure of *Egr2*-deficient cells to reconstitute the alveolar niche, we assessed the fibrotic response and subsequent repair processes in *Lyz2* ^Cre/+^.*Egr2* ^fl/fl^ mice. Notably, we did not detect differences in the degree of fibrosis or expression of key genes associated with fibrosis, including *Col3a1* and *Pdgfrb* between *Lyz2* ^Cre/+^.*Egr2* ^fl/fl^ mice and their *Egr2* ^fl/fl^ littermate controls at day 21, a time considered ‘peak’ fibrosis (Figure 8H, Supplementary Figure 10A, B). However, analysis at 6 weeks post bleomycin showed that whereas the *Egr2* ^fl/fl^ mice had largely repaired their lungs, *Lyz2* ^Cre/+^.*Egr2* ^fl/fl^ mice had defective repair evidenced by persistent fibrosis and architectural damage (Figure 8G, H, Supplementary Figure 10A). This was paralleled by elevated numbers of macrophages in the lung parenchyma (Figure 8I, Supplementary Figure 10A) and parenchymal macrophage persistence correlated with the degree of fibrosis (Supplementary Figure 10C). Furthermore, homeostasis failed to be restored in the airways. Flow cytometric analysis of BAL fluid revealed that CD45^+^ leukocytes comprised only 10% of all events in *Lyz2* ^Cre/+^.*Egr2* ^fl/fl^ mice compared with 60% in their *Egr2* ^fl/fl^ littermates (Figure 8J). The vast majority of the CD45^–^ fraction failed to express signature markers for cells of epithelial, endothelial or fibroblast origin, suggesting this may represent cellular debris, which could also be found amongst lung digests (Supplementary Figure 11A-C). This was paralleled by elevated BAL fluid protein levels and turbidity in the *Lyz2* ^Cre/+^.*Egr2* ^fl/fl^ mice compared with controls, suggesting that the inability to replenish the alveolar macrophage niche following injury was associated with the development of alveolar proteinosis (Figure 8K). Thus, loss of EGR2-dependent, monocyte-derived alveolar macrophages leads to defective tissue repair, persistent cellular damage and failed restoration of lung homeostasis.

## Discussion

Given the multifaceted role of macrophages in tissue homeostasis, inflammation and tissue repair, as well as many chronic pathologies, understanding the environmental signals and the downstream molecular pathways that govern macrophage differentiation is a key objective in the field of immunology. Here, we identify the transcription factor EGR2 as a selective and indispensable part of the tissue-specific differentiation of lung alveolar macrophages.

Our transcriptomic analysis identified EGR2 as a feature of murine lung alveolar macrophages, a finding consistent with previous studies using bulk transcriptomic analysis (5, 12) and a recent study using a similar scRNA-seq based approach (47). Our finding that EGR2 appears to represent an evolutionarily conserved transcriptional regulator is also consistent with previous studies (37, 48). While EGR2 has been implicated in controlling monocyte to macrophage differentiation in the past, these studies have often reached discrepant conclusions (20, 21). This could reflect the fact that most studies examining the role of EGR2 in monocyte-macrophage differentiation have employed *in vitro* culture systems due to the postnatal lethality of global *Egr2*-deficient mice (22, 23). By generating *Lyz2* ^Cre^.*Egr2* ^fl/fl^ and *Fcgr1* ^iCre^.*Egr2* ^fl/fl^ mice, we circumvented this lethality and demonstrated that EGR2 controls a large proportion of the alveolar macrophage ‘signature’. This is consistent with recent epigenetic analysis showing an overrepresentation of EGR motifs in the genes defining alveolar macrophages (49). Importantly, although previous work has suggested that there is redundancy between EGR family members, specifically EGR1 and EGR2, we found *Egr1* expression was unaffected by EGR2 deficiency and was unable to rescue alveolar macrophage differentiation.

Notably, if assessed simply on the basis of their CD11c^hi^CD11b^lo^ profile, the absolute number of alveolar macrophages was equivalent between adult *Egr2* ^fl/fl^ and *Lyz2* ^Cre^.*Egr2* ^fl/fl^ mice. This could explain why a recent study using an independent strain of *Lyz2* ^Cre^.*Egr2* ^fl/fl^ mice concluded that EGR2 is dispensable for macrophage differentiation (37). Alternatively, this could reflect that the majority of their studies involved *in vitro* generated, CSF1-dependent macrophages. Indeed, we found that *Egr2*-deficient monocytes matured into macrophages equally well when cultured *in vitro* with CSF-1. However, in our hands, CSF-1 led to poor upregulation of EGR2 in maturing macrophages *in vitro*. Instead, we identified GM-CSF as a potent inducer of EGR2 expression, a finding consistent with the dependence of alveolar macrophages on alveolar epithelial cell-derived GM-CSF for their development and survival (7, 8). Importantly, deficiencies in *Lyz2* ^Cre^.*Egr2* ^fl/fl^ mice did not reflect consistent differences in the expression of GM-CSF signalling molecules. EGR2 is often referred to as a feature of alternative macrophage activation on the basis that IL-4 can drive EGR2 upregulation *in vitro* in a STAT6-dependent manner (36, 37). However, we ruled out a role for IL-4 in EGR2 regulation in alveolar macrophages. Thus, the IL-4–IL-4R axis is sufficient, but not necessary, for inducing EGR2 expression *in vivo*. TGF-β also induced EGR2 and we confirmed that TGF-βR signalling is indispensable for the development of alveolar macrophages (9). If and how GM-CSF and TGF-β cooperate to promote alveolar macrophage differentiation is incompletely understood, however they both induce expression of PPAR-γ (8, 9) and *Pparg*-deficient alveolar macrophages expressed reduced EGR2 (8), suggesting EGR2 lies downstream of PPAR-γ. Whether an initial TGFβ signal is needed to induce EGR2 during alveolar macrophage development or if continual TGFβR signalling is needed to maintain EGR2 remains to be determined and will require new transgenic systems to allow inducible deletion of TGFβR. Notably, while genetic ablation of *Pparg, Csf2rb* or *Tgfbr2* leads to defects in the development and self-maintenance of alveolar macrophages, this was not replicated by *Egr2* deficiency. Thus, the EGR2-dependent programme appears to represent a discrete part of alveolar macrophage differentiation. Consistent with this, mice with myeloid or macrophage deletion of *Egr2* did not develop spontaneous alveolar proteinosis, suggesting EGR2 is redundant for regulation of surfactant. However, *Egr2*-deficient mice displayed functional deficiencies in the ability to control low dose *S. pneumoniae* infection. Although we cannot rule out the possibility that this reflects differences in the killing capacity of *Egr2*-deficient alveolar macrophages, genes encoding e.g. reactive oxygen and nitrogen species were unaffected by *Egr2* deficiency. Instead, genes encoding key pathogen recognition receptors and opsonins, were significantly downregulated in the absence of EGR2. These included MARCO and the complement component C3, both of which have been shown to be crucial for the effective elimination of *S. pneumoniae* (35, 50). Indeed, opsonisation is a critical factor in optimizing bacterial clearance by alveolar macrophages in health and disease (51). Thus, it is clear that EGR2-dependent differentiation equips alveolar macrophages with the machinery to recognise and engulf pneumococci, and this may explain the recurrent pneumonias in individuals with mutations in *EGR2* (22). In future work, it will be important to determine if this extends to other respiratory pathogens. Moreover, given that MARCO appears to define a discrete subset of CXCL2-expressing alveolar macrophages with elevated pro-inflammatory features in the context of fungal infection (52), it will be of interest to determine if all alveolar macrophages are equal in their ability to eliminate *S. pneumoniae* or if an analogous CXCL2^+^ subset with superior anti-bacterial capacity exists in this context.

Loss of alveolar macrophages is a common feature of lung inflammation or injury. Consistent with previous work (42, 53), we found that the principal mechanism of macrophage replenishment was through recruitment of BM-derived cells which mature into bona fide alveolar macrophages with time. Using *Cx3cr1*-based genetic fate mapping, we also showed that CX3CR1^+^MHCII^+^ cells with a hybrid phenotype could be found in the airways during the fibrotic phase of injury, suggesting that monocyte-derived macrophages that accumulate in the lung parenchyma following injury may replenish the alveolar macrophage niche. Although we cannot rule out that monocytes, including Ly6C^low^ monocytes (54), enter the airways during this phase to give rise to alveolar macrophages directly, the phenotype of the RFP^+^ transitional cells was more aligned with the phenotype of elicited, monocyte-derived macrophages in the parenchyma, including high levels of MHCII. Importantly, repopulation of the alveolar macrophage compartment was dependent on EGR2, with constitutive deletion of EGR2 severely blunting the engraftment of monocyte-derived cells into the alveolar macrophage niche. This contrasts with initial population of the developing alveolar niche by foetal liver-derived monocytes, where *Egr2* deficiency does not affect the development of alveolar macrophages. This could indicate differential dependence of developmentally distinct monocytes on EGR2, or the presence of compensatory pathways during development that are not present during repopulation and further work is required to fully understand this.

Interestingly, although previous work has suggested that monocyte-derived alveolar macrophages are key pro-fibrotic cells (42, 53), fibrosis appeared to develop normally in *Egr2*-deficient mice, despite the near absence of monocyte-derived alveolar macrophages. The reason for the discrepancy in our findings and those of Misharin *et al.* (42) is unclear, but it could reflect differences in the systems used. For instance, the Misharin study exploited the dependence of alveolar macrophages on Caspase-8 to impede monocyte differentiation into alveolar macrophages by using *Lyz2* ^Cre^.*Casp8* ^fl/fl^ and *Itgax* ^Cre^.*Casp8* ^fl/fl^ mice. However, deletion of Caspase-8 also affects the ability of interstitial macrophages to repopulate following depletion, meaning that *Casp8* deficiency may have wider effects on lung macrophage behaviour than disrupting the differentiation of monocyte-derived alveolar macrophages. In contrast, EGR2 expression is restricted to alveolar macrophages and deletion does not affect the reconstitution of the interstitial macrophage compartment. The location of interstitial macrophages in the parenchyma adjacent to fibroblasts and their production of the fibroblast mitogen PDGF-aa, suggests that interstitial macrophages are likely to be key to the fibrotic process (43). Indeed, depletion of interstitial macrophages using *Cx3cr1* ^Cre-ERT2^.*Rosa26* ^LSL-DTA^ mice reduces lung fibrosis (43), although as we show here, this will also target CX3CR1^+^ cells destined to become monocyte-derived alveolar macrophages. Nevertheless, our data show a clear role for monocyte-derived macrophages in tissue repair processes, as *Lyz2* ^Cre^.*Egr2* ^fl/fl^ mice failed to repair the lung after injury, a finding consistent with an older study using non-specific, clodronate-mediated depletion of lung macrophages (55) and a recent study implicating ApoE-producing, monocyte-derived alveolar macrophages in lung fibrosis resolution (56). These results may help explain the development of restrictive pulmonary disease in individuals with mutations in *EGR2* (22).

In summary, our results demonstrate that EGR2 is an evolutionarily conserved transcriptional regulator of alveolar macrophage differentiation, loss of which leads to major phenotypic, transcriptional and functional deficiencies. By identifying EGR2 as a transcriptional regulator, we have begun to dissect how common factors such as GM-CSF and TGFβ confer specificity during macrophage differentiation. Our work reveals how distinct molecular modules appear to control the homeostatic versus immune protective functions of alveolar macrophages, which may be beneficial to the host by allowing these functions to be controlled independently. Importantly, given that recent studies using human systems have proposed that alveolar macrophage maintenance in humans requires monocyte input (57, 58), EGR2 may play a particularly important role in alveolar macrophage differentiation in man. Thus, further work is required to fully understand the molecular pathways downstream of EGR2 and whether this is conserved between mouse and humans, and if EGR2 plays distinct roles in different pathological settings.

## Materials and Methods

### Study Design

We performed phenotypic, transcriptomic and functional analysis of alveolar macrophages in the context of *Egr2* deficiency to assess the features controlled by this transcription factor. Fate mapping techniques were used to assess the macrophage dynamics during bleomycin-induced injury and to test the cell intrinsic effects of *Egr2* deficiency. Infection with *Streptococcus pneumoniae* was used to assess the immune protective features of alveolar macrophages. All imaging and associated analysis was blinded. Experimental replicate details are provided in figure legends.

### Experimental Animals

Mice were bred and maintained in SPF facilities at the University of Edinburgh or University of Glasgow, UK. All experimental mice were age matched and both sexes were used throughout the study. The mice used in each experiment is documented in the appropriate figure legend. Experiments performed at UK establishments were permitted under license by the UK Home Office and were approved by the University of Edinburgh Animal Welfare and Ethical Review Body. Genotyping was performed by Transnetyx using real-time PCR. Mouse strains are detailed in Table S2.

#### Human cells

BAL fluid was obtained from patients attending the Edinburgh Lung Fibrosis Clinic. Ethical permission was granted from the NHS Lothian Research ethics board (LREC 07/S1102/20 06/S0703/53). BAL fluid cells were stained for flow cytometric analysis with antibodies listed in Table S3.

#### Tamoxifen-based fate mapping

For induction of Cre activity in *Cx3cr1* ^Cre-ERT2/+^ mice, tamoxifen was dissolved in sesame oil overnight at 50mg/ml in a glass vial and administered by oral gavage at 5mg per day for five consecutive days. In bleomycin experiments, tamoxifen was administered from d16 post bleomycin administration for 5 days. Fresh tamoxifen was prepared for each experiment.

#### Bleomycin lung injury

Bleomycin sulphate (Cayman chemicals) was prepared by first dissolving in sterile DMSO (Sigma) and further in sterile PBS at 0.66mg/ml. 8-12-week-old *Lyz2* ^Cre^.*Egr2* ^fl/fl^ and *Egr2* ^fl/fl^ littermate controls were anaesthetised with isofluorane and administered 50μl bleomycin (33μg) or vehicle control (DMSO/PBS) by oropharyngeal aspiration.

#### Streptococcus pneumoniae infection

*Lyz2* ^Cre/+^.*Egr2* ^fl/fl^ mice and *Egr2* ^fl/fl^ littermate control male mice (8–14-week-old) were anaesthetised ketamine/medetomidine and inoculated intratracheally with 50μl of PBS containing 10^4^ CFU *S. pneumoniae* (capsular type 2 strain D39). 100μl of inoculum was plated on blood agar to determine exact dose. Mice were culled 14 h later and BAL fluid collected by lavage performed using sterile PBS. 100μl of lavage fluid was cultured for bacterial growth for 24 h. The remaining lavage fluid was centrifuged at 400g for 5 mins and the resulting cells counted and prepared for flow cytometric analysis.

#### BM chimeric mice

To generate WT:*Lyz2* ^Cre^.*Egr2* ^fl/fl^ mixed chimeras, CD45.1^+^CD45.2^+^ WT mice were lethally irradiated with two doses of 5 Gy 1 hour apart before being reconstituted immediately WT (CD45.1^+^) and *Lyz2* ^Cre/+^.*Egr2* ^fl/fl^ or *Egr2* ^fl/fl^ (CD45.2^+^) bone marrow at a ratio of 1:1.Chimerism was assessed at 8 weeks after reconstitution.

#### Processing of tissues

Mice were sacrificed by overdose with sodium pentobarbitone followed by exsanguination. Mice were then gently perfused with PBS through the heart. In lung injury/fibrosis experiments, the right lobe was tied off, excised and stored in RPMI with 10% FCS on ice before being prepared for enzymatic digestion (see below). The left lung lobe was inflated with 600μl 4% PFA through an intra-tracheal canula. The trachea was tied off with thread and the lung and heart carefully excised and stored in 4% PFA overnight. Fixed lung tissue was moved to 70% ethanol before being processed for histological assessment. Right lung lobes were chopped finely and digested in pre-warmed RPMI1640 with ‘collagenase cocktail’ (0.625mg ml^−1^ collagenase D (Roche), 0.425mg ml^−1^ collagenase V (Sigma-Aldrich), 1mg ml^−1^ Dispase (ThermoFisher), and 30 U ml^−1^ DNase (Roche Diagnostics GmbH)) for 25 minutes in a shaking incubator at 37°C before being passed through a 100μm strainer followed by centrifugation at 300g for 5 mins. Erythrocytes were lysed using Red Blood Cell Lysing Buffer Hybri-Max (Sigma-Aldrich) for 2mins at room temperature, washed in FACS buffer (2% FCS/2mM EDTA/PBS) and resuspended in 5mls of FACS buffer, counted and kept on ice until staining for flow cytometry. In some experiments BAL fluid was obtained by lavaging the lungs with 0.8ml DPBS/2mM EDTA via an intra-tracheal catheter. This was repeated three times, with the first wash kept separate for analysis of BAL cytokines, turbidity and protein concentration. To obtain splenic leukocytes, spleens were chopped and digested in HBSS with 1mg/ml collagenase D for 45 mins in a shaking incubator at 37°C before being passed through a 100μm strainer followed by centrifugation at 400g for 5 mins. Erythrocytes were lysed as above. To obtain liver leukocytes, livers were perfused through the inferior vena cava with sterile PBS and liver tissue excised. Livers were then chopped finely and digested in pre-warmed collagenase ‘cocktail’ (5ml/liver) for 30 minutes in a shaking incubator at 37°C before being passed through an 100μm filter. Cells were washed twice in 50ml ice cold RPMI followed by centrifugation at 300g for 5 mins (59). Supernatants were discarded and erythrocytes were lysed. Epidermal and dermal leukocytes were isolated as described previously (60). Colonic and adipose tissue leukocytes were isolated as described previously (61–63). To obtain peritoneal leukocytes, the peritoneal cavity was lavaged with RPMI containing 2mM EDTA and 10mM HEPES (both ThermoFisher) as described previously (64). Cells were resuspended in FACS buffer, counted and kept on ice until staining for flow cytometry.

#### Flow cytometry

For analysis of unfixed cells, cells were first incubated with 0.025 μg anti-CD16/32 (2.4G2; Biolegend) for 10mins on ice to block Fc receptors and then stained with a combination of the antibodies detailed in Table S3. Where appropriate, cells were subsequently stained with streptavidin-conjugated BV650 (Biolegend). Dead cells were excluded using DAPI or 7-AAD (Biolegend) added 2mins before acquisition. When assessing intracellular markers, cells were first washed in PBS and then incubated with Zombie NIR fixable viability dye (Biolegend) for 10mins at room temperature protected from light before following the approach detailed above. Following the final wash step, cells were subsequently fixed and permeabilized using FoxP3/Transcription Factor Staining Buffer Set (eBioscience), and intracellular staining performed using antibodies detailed in Table S3. Samples were acquired using a FACS LSRFortessa or AriaII using FACSDiva software (BD) and analyzed with FlowJo software (version 9 or 10; Tree Star). Analysis was performed on single live cells determined using forward scatter height (FCS-H) versus area (FSC-A) and negativity for viability dyes. mRNA was detected by flow cytometry using PrimeFlow technology (ThermoFisher) using probes against Spp1 (AF647) according to the manufacturer’s guidelines. For staining controls in PrimeFlow analysis, the Target Probe Hybridization step was omitted with all other steps identical to samples.

#### BAL fluid analysis

The first BAL wash was centrifuged at 400g for 5mins and supernatant removed and stored at -80°C until analysis. Total protein concentrations in BAL fluid were measured by BCA Protein Assay according to the manufacturer’s instructions (ThermoFisher). Turbidity was determined following gentle mixing by diluting 25ul of sample with 75ul DPBS and measuring the optical density of 600nm and multiplying by the dilution factor. BAL cytokines were measured using 50ul undiluted sample and the Cytokine & Chemokine 26-Plex ProcartaPlex (Panel 1) assay according to manufacturer’s guidelines (ThermoFisher).

#### Lung histology

Formalin-inflated lungs were fixed overnight in 4% buffered formalin and stored in 70% ethanol. Paraffin-embedded sections of mouse lungs were stained with Masson’s trichome as per the manufacturer’s guidelines.

#### Statistics

Statistics were performed using Prism 7 (GraphPad Software). The statistical test used in each experiment is detailed in the relevant figure legend.

Details of transcriptional analysis and imaging can be found in the Supplementary Materials and Methods.

## Supplementary Material

Data File S1 Cluster defining genes in scRNA-seq (relates to Figure 1).

Data File S2 Differentially expressed genes between alveolar macrophages from *Egr2* ^fl/fl^ and *Lyz2* ^Cre/+^.*Egr2* mice (relates to Figure 3).

Data File S3 *Ex vivo* imaging of Live Precision cut Lung Slices

Data File S4 *Ex vivo* imaging of Live Precision cut Lung Slices (zoom).

Data File S5 Raw data file (Excel file).

Supplementary File

## Data Availability

All data needed to evaluate the conclusions in the paper are present in the paper or the Supplementary Materials, and RNA-seq data have been deposited in National Center for Biotechnology Information Gene Expression Omnibus public database (www.ncbi.nlm.nih.gov/geo/). Population-level RNA-seq (accession code: GSE182044) and scRNA-seq (accession code: GSE181894). *Fcgr1^iCre^* mice are available from Prof. Bernard Malissen under a material transfer agreement with the Centre d'lmmunologie de Marseille-Luminy, Aix Marseille Université. Further information and requests for resources and reagents should be directed to and will be fulfilled by the Lead Contact, Calum Bain (calum.bain@ed.ac.uk).

## One Sentence Summary

EGR2 controls alveolar macrophage function in health and disease.
