# Supplementary material for: The transcription factor EGR2 is indispensable for tissue-specific imprinting of alveolar macrophages in health and tissue repair
Source: Sci Immunol. Author manuscript; Available in PMC 2022 Jan 13. (PMC7612216; doi:10.1126/sciimmunol.abj2132)
Supplement: Supplementary File [file EMS140727-supplement-Supplementary_File.pdf]

Supplementary Information

McCowan *et al.* The transcription factor EGR2 is indispensable for tissue-specific imprinting of alveolar macrophages in health and tissue repair

Supplementary Methods

Transcriptional analysis.
Immunofluorescence imaging.
Image analysis
Live Precision Cut Lung Slices (PCLS) imaging.
4D images analysis.

Supplementary Figures

Figure S1: Gating strategy for and cluster annotation of scRNA-seq data (associated with Figure 1).
Figure S2: Validation of EGR2 expression by mouse and human alveolar macrophages (associated with Figure 2).
Figure S3: Effects of *Egr2* deficiency on macrophages in brain, spleen and adipose tissue (associated with Figure 2).
Figure S4: Gating strategy for alveolar macrophage purification and representative purity (associated with Figure 3).
Figure S5: Analysis of alveolar macrophage motility and morphodynamics (associated with Figure 3).
Figure S6: Analysis of *Fcgr1*$^{iCre/+}$.*Egr2*$^{fl/fl}$ and *Il4ra*$^{-/-}$ mice (associated with Figure 4 & 5).
Figure S7: EGR2 expression in context of *Strepococcus pneumoniae* infection (associated with Figure 4).
Figure S8: Validating the use of the *Cx3cr1*$^{Cre\text{-}ERT2/+}$.*Rosa26*$^{LSL\text{-}RFP/+}$ fate mapping model (associated with Figure 7).
Figure S9: Effects of *Egr2* deficiency on parenchymal myeloid cells during bleomycin induced fibrosis (associated with Figure 8).
Figure S10: Assessment of the effects of *Egr2* deficiency on lung injury and fibrosis (associated with Figure 8).
Figure S11: Phenotypic characterisation of the airway and parenchymal CD45^{-} fraction during resolution of bleomycin induced lung injury (associated with Figure 8).

Supplementary Tables

Table S1: Gene ontology analysis of differentially expressed genes between alveolar macrophages from *Egr2*$^{fl/fl}$ and *Lyz2*$^{Cre/+}$.*Egr2* mice (relates to Figure 3).
Table S2: List of mouse strains
Table S3: List of antibodies
Table S4: List of primers

Supplementary Material and Methods

***Transcriptional Analysis*.**

qPCR: Real-time PCR assays for the detection of mRNAs were performed using Light Cycler System (Roche) and 384-Well Reaction Plates (Roche). Primer sequences detailed in **Table S4**. Reactions were performed using SYBR Green System (LightCycler® 480 SYBR Green I Master) according to the manufacturer protocol. 1ul of cDNA (1:50 dilution) were used per sample in a total reaction volume of 10uL. The temperature profile used was as follows: pre-denaturation 5 min at 95°C and then 45 cycles of denaturation for 10s at 95°C, annealing 10s at 60°C, elongation 10s at 72°C. Fluorescence data collection was performed at the end of each elongation step. All samples were tested in duplicates and nuclease free water was used as a non-template control. The relative change was calculated using the $2^{-\Delta\Delta Ct}$ method *(65)*, normalized to *Ppia*.

Bulk sequencing: Alveolar macrophages were FACS-purified from lung digests from unmanipulated female *Lyz2*^{Cre}.*Egr2*$^{fl/fl}$ mice or *Egr2*$^{fl/fl}$ controls. For each population, 25,000 cells were sorted into 500ml RLT buffer (Qiagen) and snap frozen on dry ice. RNA was isolated using the RNeasy Plus Micro kit (Qiagen). RNA samples were quantified using Qubit 2.0 Fluorometer (Life Technologies, Carlsbad, CA, USA) and RNA integrity was checked with 2100 TapeStation (Agilent Technologies, Palo Alto, CA, USA). SMART-Seq v4 Ultra Low Input Kit for Sequencing was used for full-length cDNA synthesis and amplification (Clontech, Mountain View, CA), and Illumina Nextera XT library was used for sequencing library preparation. Briefly, cDNA was fragmented and adaptor was added using Transposase, followed by limited-cycle PCR to enrich and add index to the cDNA fragments. The final library was assessed with Qubit 2.0 Fluorometer and Agilent TapeStation. The sequencing libraries were multiplexed and clustered on two lanes of a flowcell. After clustering, the flowcell were loaded on the Illumina HiSeq instrument according to manufacturer's instructions. The samples were sequenced using a 2x150 Paired End (PE) configuration. Image analysis and base calling were conducted by the HiSeq Control Software (HCS) on the HiSeq instrument. Raw sequence data (.bcl files) generated from Illumina HiSeq were be converted into fastq files and de-multiplexed using Illumina bcl2fastq v. 2.17 program. One mis-match was allowed for index sequence identification. After demultiplexing, sequence data was checked for overall quality and yield. Then, sequence reads were trimmed to remove possible adapter sequences and nucleotides with poor quality using Trimmomatic v.0.36. The trimmed reads were mapped to the *Mus musculus* mm10 reference genome available on ENSEMBL using the STAR aligner v.2.5.2b. The STAR aligner uses a splice aligner that detects splice junctions and incorporates them to help align the entire read sequences. BAM files were generated as a result of this step. Unique gene hit counts were calculated by using featureCounts from the Subread package v.1.5.2. Only unique reads that fell within exon regions were counted. After extraction of gene hit counts, the gene hit counts table was used for downstream differential expression analysis. Using DESeq2, a comparison of gene expression between the groups of samples was performed. The Wald test was used to generate p-values and Log2 fold changes. Genes with adjusted p-values < 0.05 and absolute log2 fold changes > 1 were called as differentially expressed genes for each comparison.

scRNA-seq: CD11c$^+$ and CD11b$^+$ cells, excluding Ly6G$^+$ and SiglecF$^+$CD11b$^+$ eosinophils, were FACS-purified from unmanipulated an adult *Rag1*$^{-/-}$ mouse. Single cells were processed through the Chromium Single Cell Platform using the Chromium Single Cell 3' Library and Gel Bead Kit V2 and the Chromium Single Cell A Chip Kit (both 10X Genomics) as per the manufacturer's protocol *(66)*. Briefly, single myeloid cells were purified by FACS into PBS/2% FBS, washed twice and cell number measured using a Bio-Rad TC20 Automated Cell Counter (BioRad). Approximately 10,000 cells were

loaded to each lane of a 10X chip and partitioned into Gel Beads in Emulsion containing distinct barcodes in the Chromium instrument, where cell lysis and barcoded reverse transcription of RNA occurred, followed by amplification, fragmentation and 5' adaptor and sample index attachment. Libraries were sequenced on an Illumina HiSeq 4000. For analysis, Illumina BCL sequencing files were demultiplexed using 10x Cell Ranger (version 2.1.1; https://www.10xgenomics.com; ‘cellranger_mkfastq’). Resultant FASTQ files were fed into ‘cellranger_count’ with the transcriptome ‘refdata-cellranger-mm10-1.2.0’ to perform genome alignment, filtering, barcode counting and UMI counting. Downstream QC, clustering and gene expression analysis was performed using the Seurat R package (V3; R version 4.0.2) following the standard pre-processing workflow *(67)*. Cells were filtered on QC covariates used to identify nonviable cells or doublets: number of unique genes per cell (nFeatureRNA >200 & <4000); percentage mitochondrial genes (<20%). Data for resultant 3936 cells were normalized and scaled prior to PCA analysis. Unsupervised clustering based on the first 20 principal components of the most variably expressed genes was performed using a KNN graph-based approach and resultant clusters visualised using the Uniform Manifold Approximation and Projection (UMAP) method. Differential gene expression analysis was used to identify genes expressed by each cell cluster relative to all others, using the nonparametric Wilcoxon rank-sum test and p-value threshold of <0.05. Canonical cell phenotypes were assigned to individual clusters based on the expression of known landmark gene expression profiles.

Publicly available datasets were downloaded from the COVID-19 Cell Atlas *(17-19)* to perform *in silico* analysis of EGR2 expression in human tissue macrophages. Data were pre-processed and merged using the Seurat R package (V3; R version 4.0.2) following standard methods. Macrophages were extracted based on the expression of C1QA > 0 to compare expression of EGR2 in different human tissue settings.

Immunofluorescence imaging. Imaging was performed as described recently *(68)*. Briefly, samples were permeabilized and blocked for 20min in PBS/Neutral goat serum (NGS) 10%/BSA1%/TritonX-100 (Tx100) 0.3%/Azide 0.05% at 37 °C and stained with 150 µl rabbit anti-CD68 Ab (Polyclonal, ab125212, abcam, 1/200) diluted in PBS/ NGS10%/BSA1%/ TX-100 0.3%/Azide 0.05% for 20min. Samples were washed 3 times with PBS/BSA1%/TX-100 0.1%/Azide 0.05% before adding 150 µl of a solution containing DAPI (1/10000), aSMA-Cy3 (clone 1A4, Sigma, 1/1000), anti-rabbit-AF488 (polyclonal, A-21206 ThermoFisher) diluted in PBS/ NGS10%/BSA1%/ TX-100 0.3%/Azide 0.05% for 1h. Samples were washed 3 times with PBS/BSA1%/TX-100 0.1%/Azide 0.05% and 2 times in PBS. Finally slides were mounted with Vectashield (Vector Laboratories, H-1700). Images were acquired with a Zeiss LSM 880 NLO multiphoton microscope (Carl Zeiss, Oberkochen, Germany) equipped with a 32 channel Gallium arsenide phosphide (GaAsP) spectral detector using 20×/1 NA water immersion objective lens. Samples were excited with a tunable laser (680–1300 nm) set up at 1000 nm and signal was collected onto a linear array of the 32 GaAsp detectors in lambda mode with a resolution of 8.9 nm over the visible spectrum. Spectral images were then unmixed with Zen software (Carl Zeiss) using references spectra acquired from unstained tissues (tissue autofluorescence and second harmonic generation) or beads labelled with Cy3- or AF488-conjugated antibodies.

Image analysis. Fluorescence images were analysed with QuPath *(69)*. Full lung section was annotated using the “simple tissue detection” tool and non-pulmonary tissue (trachea, heart tissue) were manually removed from the annotation. In order to refine the analysis, “Pixel classification” was used to segment lung regions of interest. Briefly, software was trained to recognize the different regions using fluorescence (aSMA, SHG and autofluorescence) and texture (all available) features from example images. 2-3 example areas per regions of interest were annotated for each lung to train the pixel classifier. The following regions were analysed: (1) normal lung parenchyma/alveolar tissue, (2)

pathologic/fibrotic tissue and (3) collagen rich areas: perivascular/(peri)bronchial spaces + pleura were segmented to avoid false fibrotic region detection. Macrophages were detected using the "Positive cell detection" tool and were expressed as the number $DAPI^+$ $CD68^+$ cells/mm^2 of analysed region (full section or regions of interest). Fibrosis was defined as percentage of full section with fibrotic features. All fibrosis scoring and macrophage quantification was performed in a blinded fashion.

Live Precision Cut Lung Slices (PCLS) imaging. Live PCLS procedure was adapted from (https://doi.org/10.1101/680611). Mice were humanely killed by i.p. injection of sodium-pentobarbital, a small incision was made in the trachea and a customized blunted 22G needle was inserted. Subsequently, 1 mL of 2% low-melting point agarose was instilled slowly through the needle. Excised lungs were placed in 10% FBS/RPMI. Lungs were sliced into 300µm thick sections on a vibratome and stained with directly conjugated Ab (**Table S3**) in complete medium (phenol-red free DMEM substituted with 10% FBS) for 20 minutes at 37°C. Slices were imaged on a Zeiss LSM880 confocal microscope in a full incubation chamber at 37°C with 5% CO2. Lung slices were imaged for 2x11min with z-stacks of 22.5um. Acquisition was performed with a 32 channel Gallium arsenide phosphide (GaAsP) spectral detector using 20× objective. Samples were excited simultaneously with 405, 488, 561 and 633 laser lines and signal was collected onto a linear array of the 32 GaAsp detectors in lambda mode with a resolution of 8.9 nm over the visible spectrum. Spectral images were then unmixed with Zen software (Carl Zeiss) using reference spectra acquired from unstained tissues (tissue autofluorescence) or beads labelled with single fluorophores.

4D images analysis. Timelapse images analysis and visualization was performed using Imaris (Bitplane). Neutrophils and macrophages were segmented and tracked using the 'surface' tool using either CD11b and Ly6G fluorescence intensities (neutrophils) or CD11c (macrophages). All surfaces were checked manually to avoid any false detections. Cell behavior was determined using the track displacement length (indicating cell mobility) and the standard deviation of cell sphericity (indicating changes in cell shape over time).

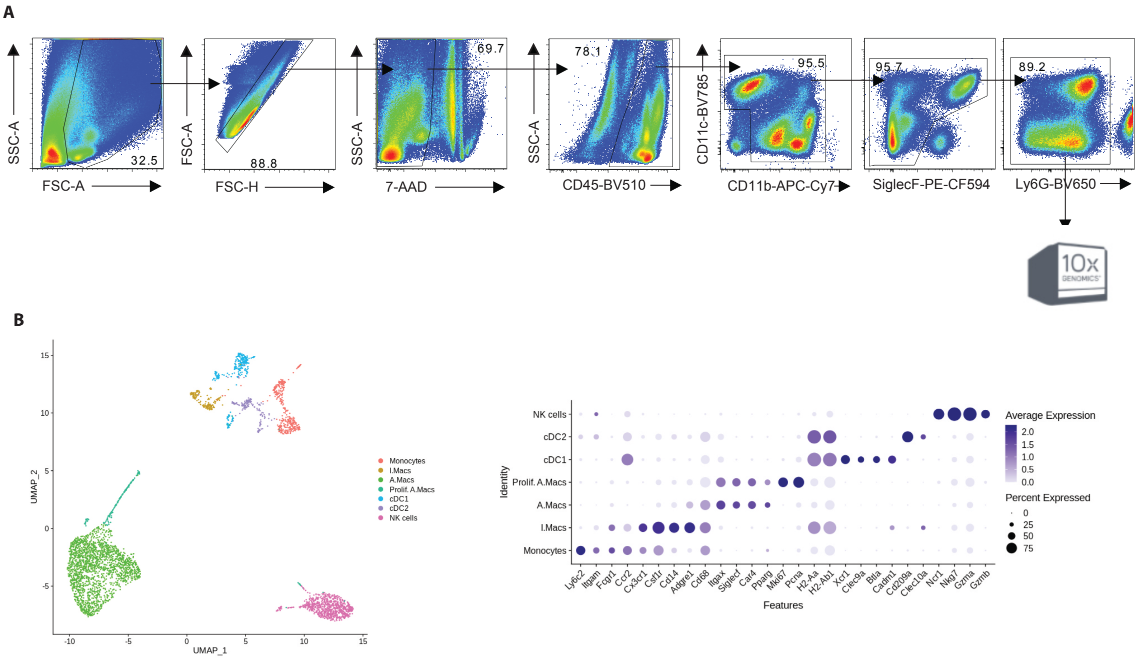

Figure S1: Gating strategy for and cluster annotation of scRNA-seq data

A. Gating strategy used for the purification of mononuclear phagocytes for scRNA-seq analysis using the 10X Genomics platform.

B. UMAP dimensionality reduction analysis of non-granulocyte reveals seven clusters of cells in lungs of adult *Rag1*$^{-/-}$ mice (*left*). Right, expression of canonical markers to validate annotations.

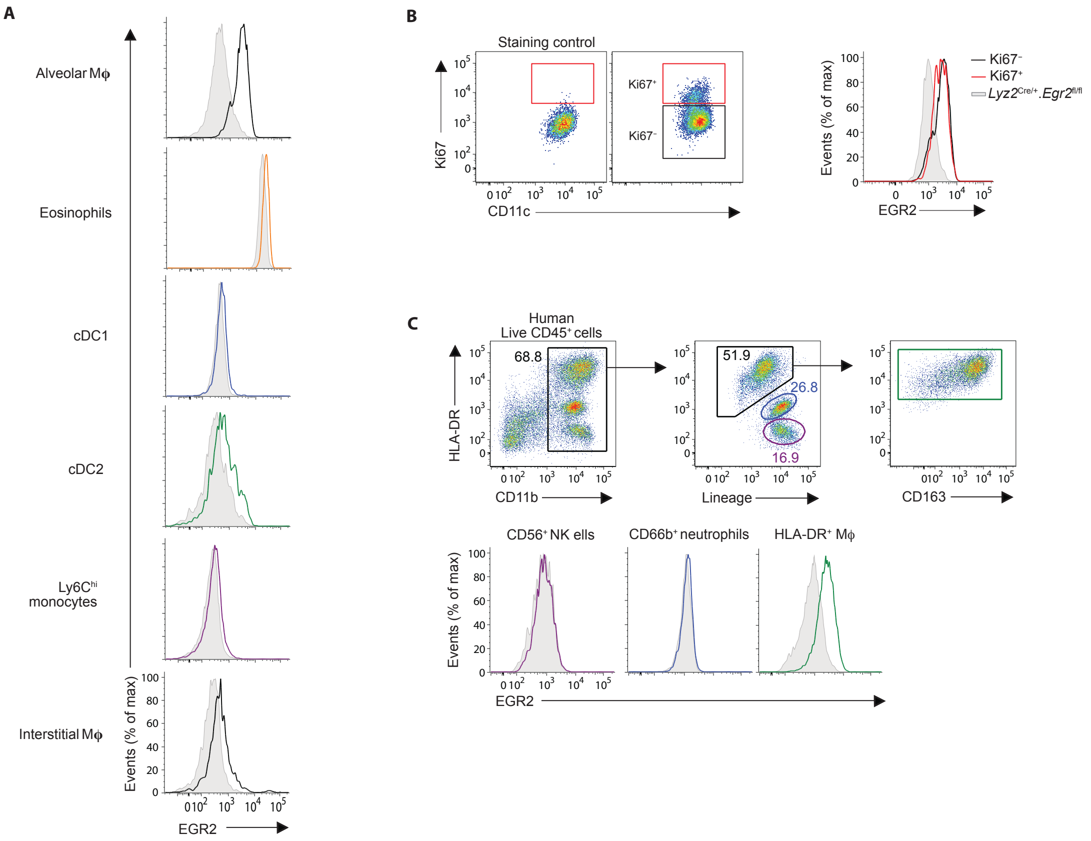

Figure S2: Validation of EGR2 expression by mouse and human alveolar macrophages

A. Expression of EGR2 by indicated myeloid cells in the lungs of *Egr2*$^{fl/fl}$ mice [coloured line] or *Lyz2*$^{Cre/+}$.*Egr2*$^{fl/fl}$ (Cre^{+}) mice [shaded histogram]. Data from one of at least three independent experiments. Alveolar macrophage histogram is duplicated from Figure 1 for reference.

B. Representative expression of EGR2 by Ki67-defined alveolar macrophages from *Egr2*$^{fl/fl}$ mice [coloured lines] or *Lyz2*$^{Cre/+}$.*Egr2*$^{fl/fl}$ mice [shaded histogram]. Data from one of at least three independent experiments.

C. Gating strategy for the identification of alveolar macrophages and granulocytes (lineage^{+}) in the BAL fluid from an individual with idiopathic pulmonary fibrosis (IPF) and expression of EGR2 by the indicated populations. Data is representative of three individual patients.

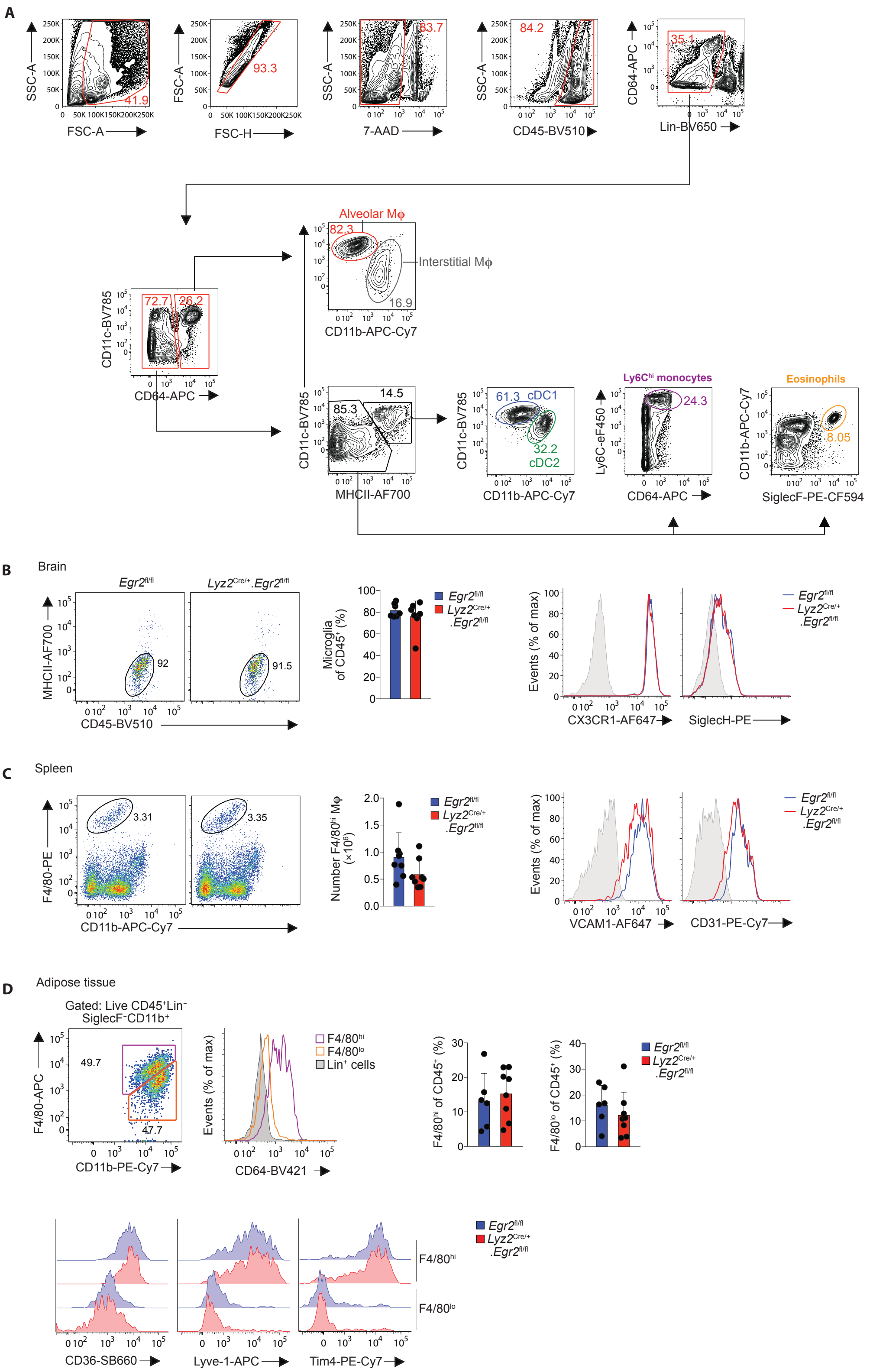

Figure S3: Effects of *Egr2* deficiency on macrophages in brain, spleen and adipose tissue

A. Gating strategy for the identification of distinct myeloid cell subsets in the lungs of *Egr2*$^{fl/fl}$ or *Lyz2*$^{Cre/+}$.*Egr2*$^{fl/fl}$ mice.

B. Representative expression of CD45 and MHCII by CD11b^{+}Ly6C^{-}lineage^{-} cells from brains of adult *Egr2*$^{fl/fl}$ or *Lyz2*$^{Cre/+}$.*Egr2*$^{fl/fl}$ mice. Graph shows the frequency of CD45^{lo}MHCII^{-} microglia of CD45^{+}

cells. Histograms show representative expression of CX3CR1 and SiglecH by CD45^{lo}MHCII^{-} microglia. Shaded histograms represent FMO controls. Data pooled from three independent experiments.

C. Representative expression of F4/80 and CD11b by lineage^{-} cells from spleens of adult *Egr2*$^{fl/fl}$ or *Lyz2*$^{Cre/+}$.*Egr2*$^{fl/fl}$ mice. Graph shows the absolute number of F4/80^{hi} macrophages per spleen in each group. Histograms show representative expression of VCAM1 and CD31 by F4/80^{hi} macrophages. Shaded histograms represent FMO controls. Data pooled from three independent experiments.

D. Representative expression of F4/80 and CD11b by lineage^{-} cells from gonadal adipose tissue of adult *Egr2*$^{fl/fl}$ mice to define mononuclear phagocyte subsets and their expression of CD64. Graphs shows the frequencies of F4/80-defined subsets in adult *Egr2*$^{fl/fl}$ or *Lyz2*$^{Cre/+}$.*Egr2*$^{fl/fl}$ mice. Histograms show representative expression of CD36, Lyve-1 and Tim4 by F4/80^{hi} macrophages. Shaded histograms represent FMO controls. Data pooled from three independent experiments.

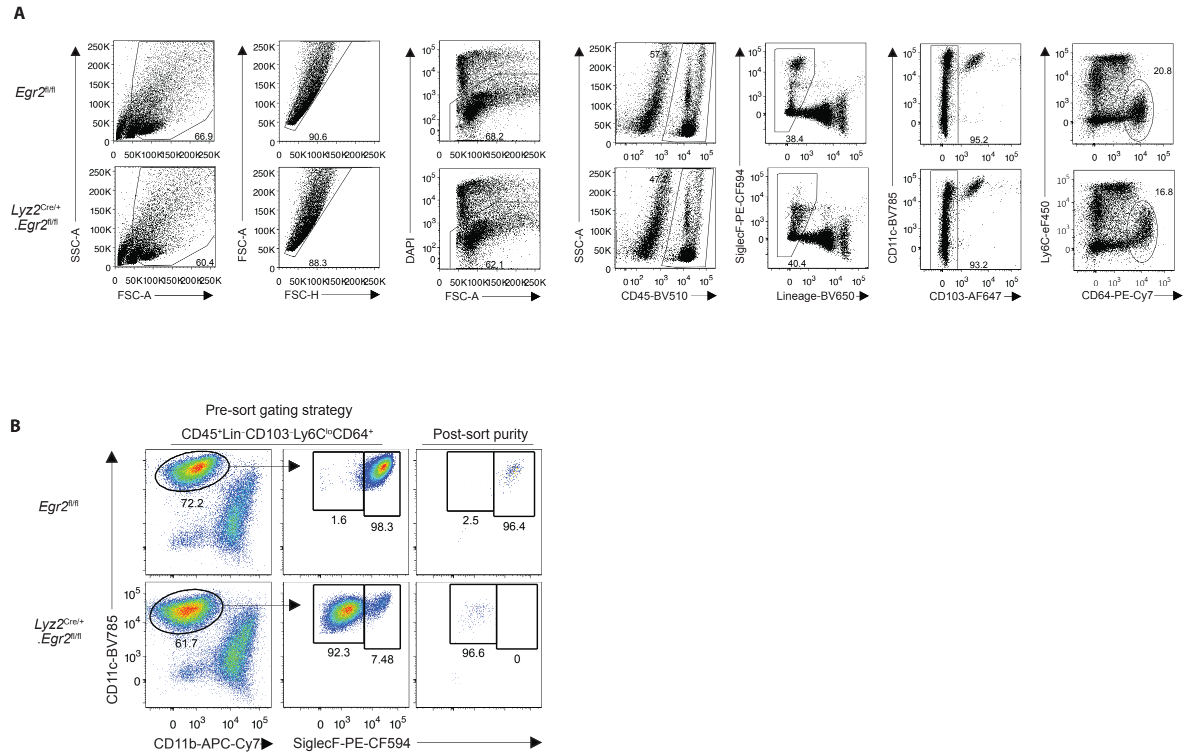

Figure S4: Gating strategy for alveolar macrophage purification and representative purity

A. Gating strategy for the FACS purification of alveolar macrophages from adult *Egr2*$^{fl/fl}$ or *Lyz2*$^{Cre/+}$.*Egr2*$^{fl/fl}$ mice for bulk RNA-seq.

B. Representative post-sort purity of alveolar macrophages in each group.

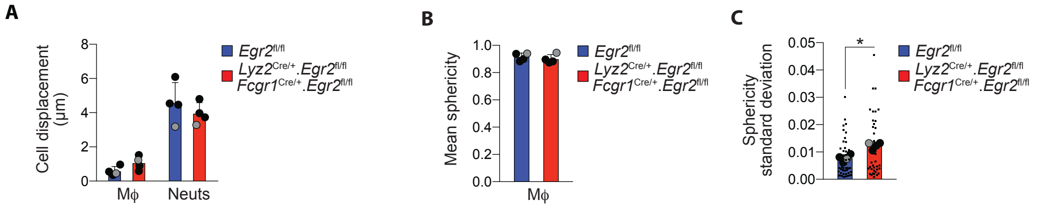

Figure S5: Analysis of alveolar macrophage motility and morphodynamics

A. Mean displacement length of alveolar macrophages and neutrophils in lungs from *Lyz2*$^{Cre/+}$.*Egr2*$^{fl/fl}$ mice (black symbols) or *Fcgr1*$^{iCre/+}$.*Egr2*$^{fl/fl}$ mice (grey symbol) or *Egr2*$^{fl/fl}$ littermate controls over 11mins in Precision Lung slices *ex vivo* – see **Data file S3** and **S4** for representative timelapse images.

B. Mean sphericity of alveolar macrophages calculated from **A**.

C. Standard deviation of alveolar macrophage sphericity over time calculated from **A**. All individual macrophages are displayed (small symbols) together with mean values for individual mice (large symbols). *$p<0.05$ (Mann-Whitney test).

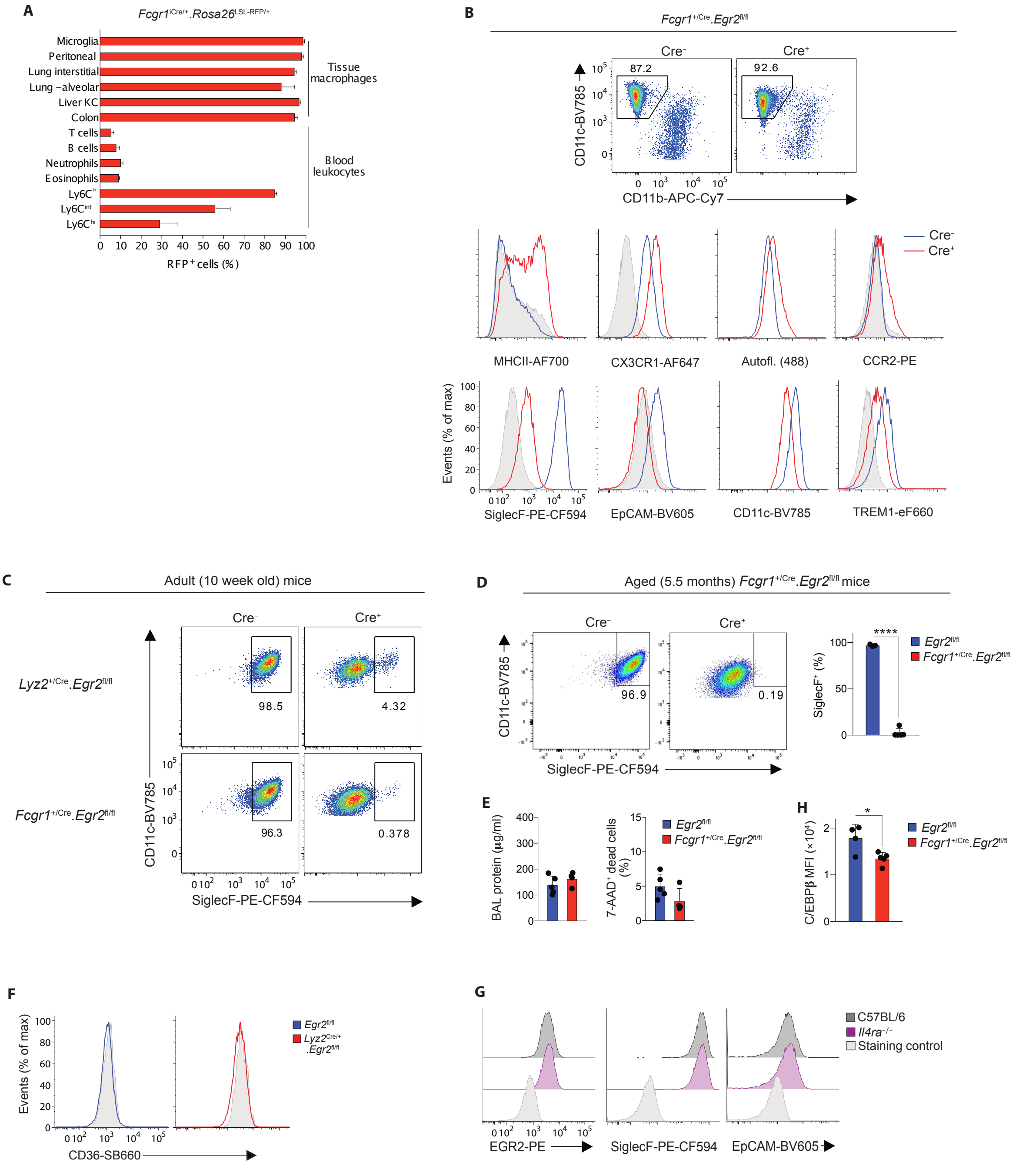

Figure S6: Analysis of *Fcgr1*$^{\text{iCre/+}}$.*Egr2*$^{\text{fl/fl}}$ and *Il4ra*$^{-/-}$ mice

A. Expression of RFP by indicated leukocytes obtained from adult *Fcgr1*$^{\text{iCre/+}}$.*Rosa26*$^{\text{LSL-RFP/+}}$ mice. Data from one of two independent experiments performed with 2-3 mice per tissue.

B. Representative expression of CD11c and CD11b by lineage$^-$Ly6C$^-$CD64$^+$ cells amongst lung tissue isolates from *Egr2*$^{\text{fl/fl}}$ mice or *Fcgr1*$^{\text{iCre/+}}$.*Egr2*$^{\text{fl/fl}}$ littermates. Data are from one experiment of two performed.

C. Representative expression of CD11c and SiglecF by lineage$^-$Ly6C$^-$CD64$^+$CD11c$^+$CD11b$^-$ alveolar macrophages from 8 week old *Egr2*$^{\text{fl/fl}}$ mice or *Lyz2*$^{\text{Cre/+}}$.*Egr2*$^{\text{fl/fl}}$ littermates and an independent colony of *Egr2*$^{\text{fl/fl}}$ mice or *Fcgr1*$^{\text{iCre/+}}$.*Egr2*$^{\text{fl/fl}}$ littermates. Data are from one experiment of two performed.

D. Representative expression of CD11c and SiglecF by lineage$^-$Ly6C$^-$CD64$^+$CD11c$^+$CD11b$^-$ alveolar macrophages from 5.5 month old *Egr2*$^{\text{fl/fl}}$ mice or *Fcgr1*$^{\text{iCre/+}}$.*Egr2*$^{\text{fl/fl}}$ littermates. Graph shows the

frequency of SiglecF^{+} macrophages of all alveolar macrophages in each group. Data are from one experiment with 3 (*Egr2*$^{fl/fl}$) or 5 (*Fcgr1*$^{iCre/+}$.*Egr2*$^{fl/fl}$) mice per group. ****$p<0.0001$ (unpaired Student's *t* test).

E. Protein levels and frequency of 7-AAD^{+} dead cells in the BAL fluid of *Egr2*$^{fl/fl}$ mice or *Fcgr1*$^{iCre/+}$.*Egr2*$^{fl/fl}$ littermates at 5.5 months months of age. Symbols represent individual mice and error is s.d.. Data represent 3-4 mice per group from one experiment. Data are from one experiment with 5 (*Egr2*$^{fl/fl}$) or 5 (*Fcgr1*$^{iCre/+}$.*Egr2*$^{fl/fl}$) mice per group.

F. Representative expression of CD36 by by alveolar macrophages amongst lung digests from adult *Egr2*$^{fl/fl}$ mice or *Lyz2*$^{Cre/+}$.*Egr2*$^{fl/fl}$ littermates.

G. Expression of EGR2, SiglecF and EpCAM by alveolar macrophages amongst lung digests from adult C57BL/6 or *Il4ra*$^{-/-}$ mice. Data represent 4 mice per group from one experiment.

H. Expression of C/EBPβ (MFI) by lineage^{-}Ly6C^{-}CD64^{+}CD11c^{+}CD11b^{-} alveolar macrophages from 5.5 month old *Egr2*$^{fl/fl}$ mice or *Fcgr1*$^{iCre/+}$.*Egr2*$^{fl/fl}$ littermates. Data are from one experiment with 3 (*Egr2*$^{fl/fl}$) or 5 (*Fcgr1*$^{iCre/+}$.*Egr2*$^{fl/fl}$) mice per group. *$p<0.05$ (unpaired Student's *t* test).

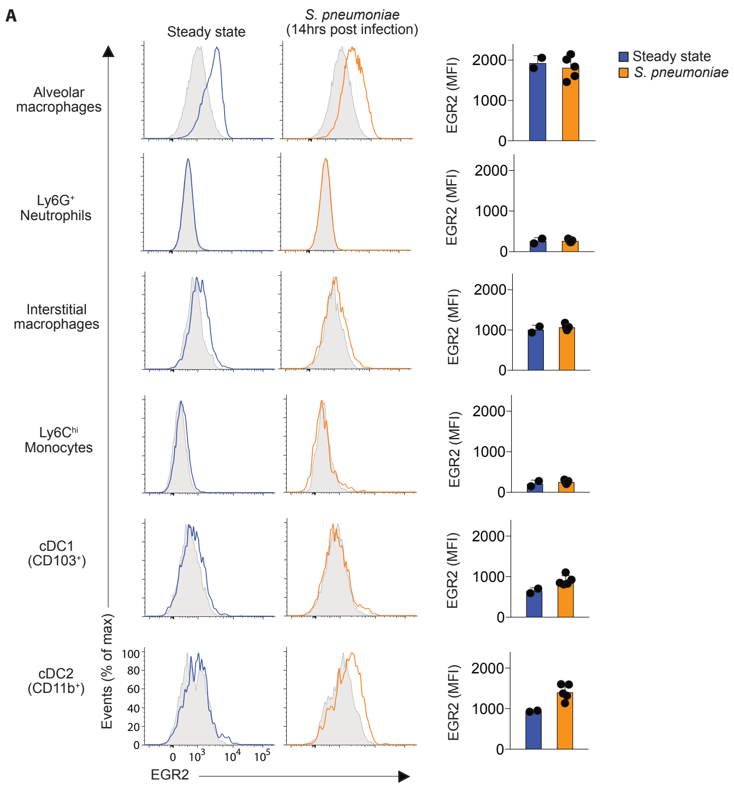

Figure S7: EGR2 expression in context of *Strepococcus pneumoniae* infection

A. Expression of EGR2 by indicated leukocytes obtained from adult *Egr2*$^{fl/fl}$ mice infected with 10^4 CFU *Streptococcus pneumoniae* 14hrs earlier (orange lines) or left uninfected (blue lines). Shaded histograms represent EGR2 expression by indicated cells from *Lyz2*$^{Cre/+}$.*Egr2* mice. Graphs show the mean fluorescence intensity (MFI) of EGR2 expression by the indicated subsets at steady state or after infection. Data are from one experiment with 2 (steady state) or 5 (*S. pneumoniae*).

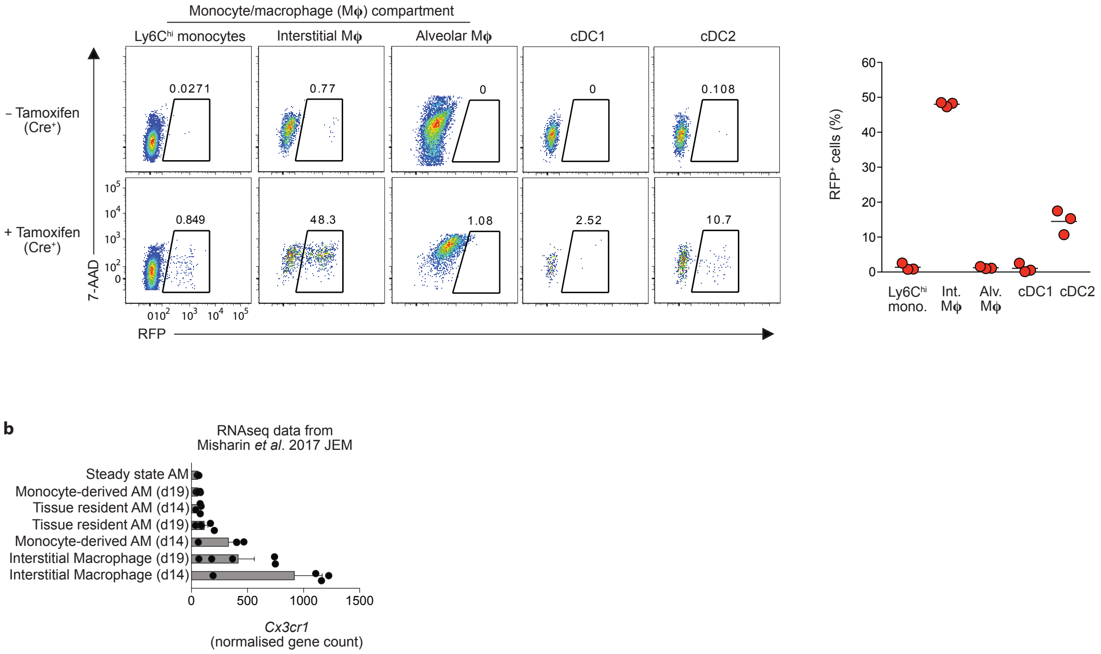

Figure S8: Validating the use of the *Cx3cr1*$^{\text{Cre-ERT2/+}}$.*Rosa26*$^{\text{LSL-RFP/+}}$ fate mapping model

A. Expression of RFP by indicated myeloid cells obtained from adult *Cx3cr1*$^{\text{Cre-ERT2/+}}$.*Rosa26*$^{\text{LSL-RFP/+}}$ mice 24hrs after the final dose of tamoxifen. Mice were administered 5mg tamoxifen by oral gavage for 5 consecutive days. Graph shows the frequency of RFP^{+} amongst each myeloid population. Data are from one of three independent experiments performed.

B. Expression of *Cx3cr1* by the indicated populations in steady state or the fibrotic phase of bleomycin-induced fibrosis. Data obtained from *(40)*.

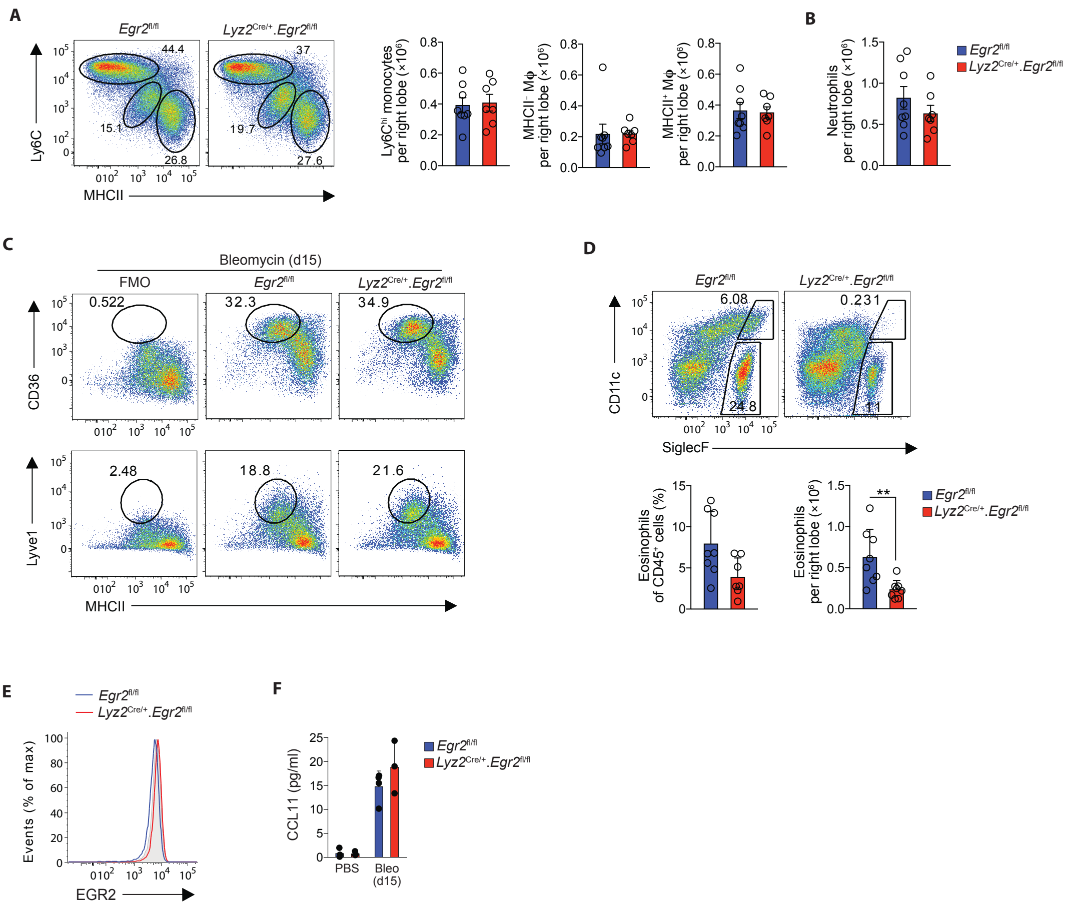

Figure S9: Effects of *Egr2* deficiency on parenchymal myeloid cells during bleomycin induced fibrosis

A. Representative expression of Ly6C and MHCII by lineage^{-}CD64^{+}CD11c^{var}CD11b^{+} cells obtained from tissue digests of lungs obtained from adult *Egr2*$^{fl/fl}$ mice or *Lyz2*$^{Cre/+}$.*Egr2*$^{fl/fl}$ littermates 15 days after the administration of bleomycin. Graphs show the absolute number of Ly6C^{hi} monocytes and MHCII-defined macrophages per right lung lobe. **p<0.01, unpaired Student's *t* test.

B. Absolute number of Ly6G^{+} neutrophils per right lung lobe of mice in **A**.

C. Representative expression of CD36 and Lyve-1 by Ly6C^{-} interstitial macrophages in lungs of adult *Egr2*$^{fl/fl}$ mice or *Lyz2*$^{Cre/+}$.*Egr2*$^{fl/fl}$ littermates 15 days after the administration of bleomycin.

D. Representative expression of CD11c and SiglecF by live CD45^{+} leukocytes obtained from tissue digests of lungs obtained from adult *Egr2*$^{fl/fl}$ mice or *Lyz2*$^{Cre/+}$.*Egr2*$^{fl/fl}$ littermates 15 days after the administration of bleomycin. Graphs show the frequency and absolute number of SiglecF^{+}CD11c^{lo} eosinophils per right lung lobe. **p<0.01 (unpaired Student's *t* test).

E. Representative expression of EGR2 by CD11c^{lo}SiglecF^{+} eosinophils 15 days after the administration of bleomycin. Symbols represent individual mice and data are pooled from two independent experiments with 8 (*Egr2*$^{fl/fl}$) or 7 (*Lyz2*$^{Cre/+}$.*Egr2*$^{fl/fl}$) mice per group.

F. Levels of CCL11 in BAL fluid from adult *Egr2*$^{fl/fl}$ mice or *Lyz2*$^{Cre/+}$.*Egr2*$^{fl/fl}$ littermates 15 days after the administration of bleomycin or PBS controls. Symbols represent individual mice with 3-4 mice per group.

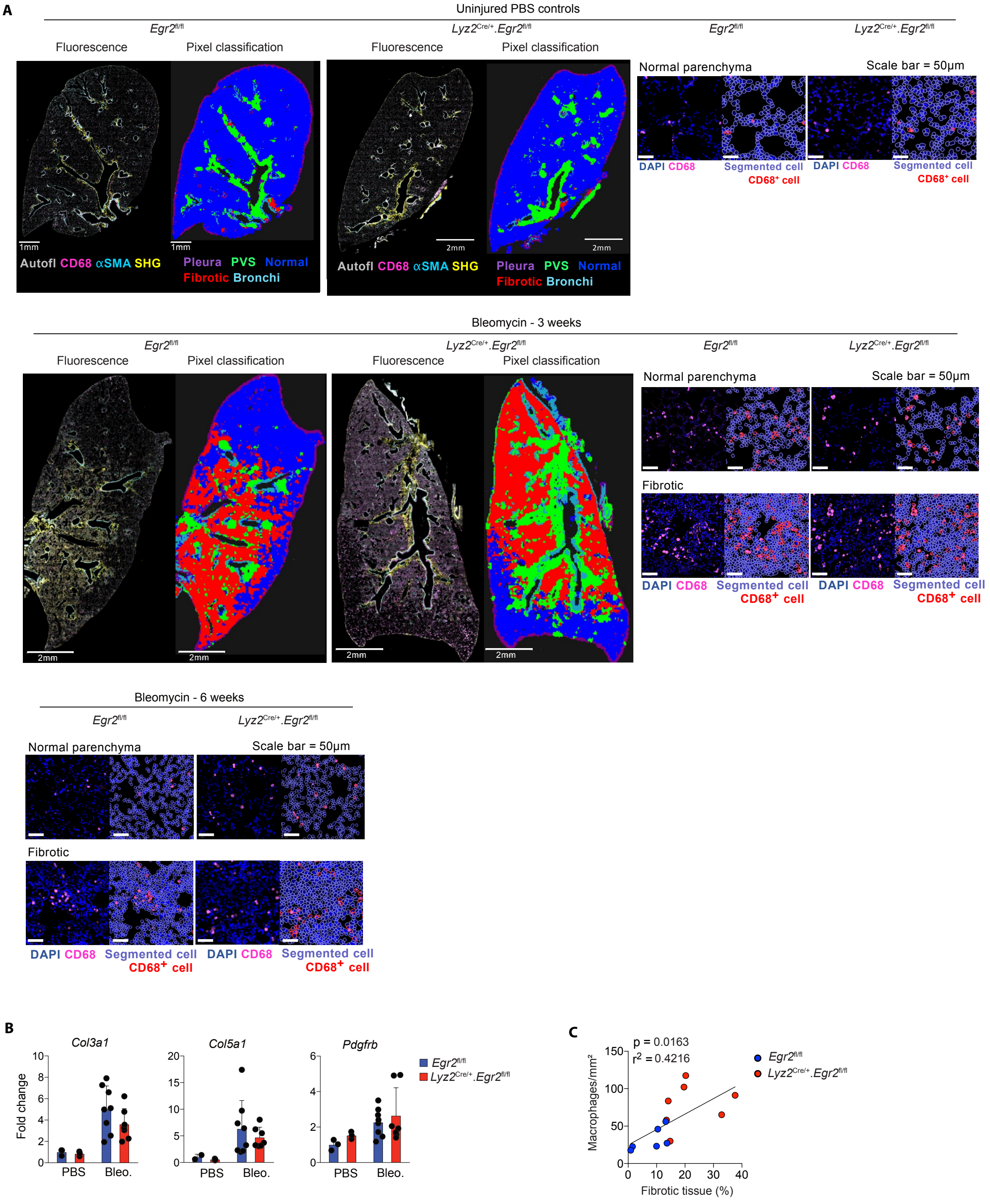

Figure S10: Assessment of the effects of *Egr2* deficiency on lung injury and fibrosis
A. 2-photon fluorescence imaging analysis of lung tissue from adult *Egr2*$^{fl/fl}$ mice or *Lyz2*$^{Cre/+}$.*Egr2*$^{fl/fl}$ mice administered PBS (uninjured) or bleomycin 3 or 6 weeks earlier. Sections were stained for CD68, αSMA and DAPI. Autofluorescence is depicted in grey and collagen detected by second harmonic generation (SHG). Pixel classification was used to segment lung regions of interest: (1) normal lung parenchyma/alveolar tissue, (2) pathologic/fibrotic tissue and (3) collagen rich areas (perivascular/bronchial spaces and pleura) were segmented to avoid false fibrotic region detection. *Right,* CD68^{+} macrophage segmentation in lung parenchyma and fibrotic areas.

B. Quantitative RT-PCR analysis of *Col3a1* and *Pdgfrb* mRNA in tissue homogenates from lungs of uninjured adult *Egr2*$^{fl/fl}$ mice or *Lyz2*$^{Cre/+}$.*Egr2*$^{fl/fl}$ littermates or mice administered bleomycin 14 days earlier. Data are pooled from two independent experiments with 3 (PBS groups), 7 (*Lyz2*$^{Cre/+}$.*Egr2*$^{fl/fl}$) or 8 (*Egr2*$^{fl/fl}$) mice per bleomycin group.

C. Correlation between the number of macrophages (per mm^2) in the lung section and lung fibrosis (% of tissue). Linear regression: R^2 = 0,4216, p=0.0163. n=7 (*Lyz2*$^{Cre/+}$.*Egr2*$^{fl/fl}$) and 8 (*Egr2*$^{fl/fl}$) mice.

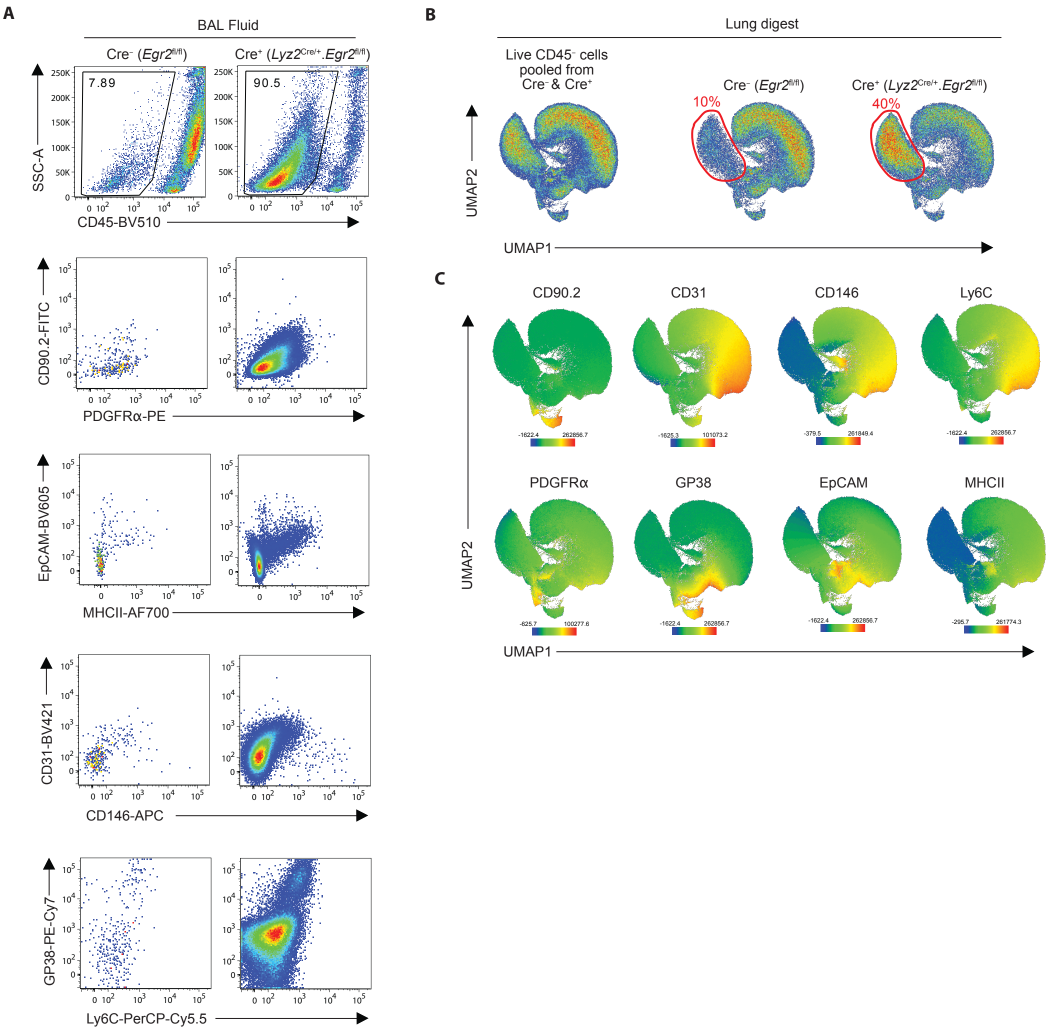

Figure S11: Phenotypic characterisation of the airway and parenchymal CD45$^-$ fraction during resolution of bleomycin induced lung injury

A. Representative expression of CD45, CD90.2, PDGFRα, EpCAM, MHCII, CD31, CD146, GP38 and Ly6C by events present in the BAL fluid of adult *Egr2*$^{fl/fl}$ mice or *Lyz2*$^{Cre/+}$.*Egr2*$^{fl/fl}$ littermates administered bleomycin 6 weeks earlier.

B. UMAP analysis of CD45$^-$ cells pooled from adult *Egr2*$^{fl/fl}$ mice or *Lyz2*$^{Cre/+}$.*Egr2*$^{fl/fl}$ littermates 6 weeks after bleomycin-induced injury (*left panel*). Right panel show the contribution of cells deriving from *Egr2*$^{fl/fl}$ mice and *Lyz2*$^{Cre/+}$.*Egr2*$^{fl/fl}$ mice to each cluster. Heatmap plots showing the relative expression of the indicated markers by clusters in **B**.

Table S1: Gene ontology analysis of differentially expressed genes between alveolar macrophages from *Egr2*$^{fl/fl}$ and *Lyz2*$^{Cre/+}$*.Egr2* mice (relates to Figure 3).

Process_name	Significant_genes_count	Total_genes_count	%_significant_genes	P-value	Padj-value
GO:0006935~chemotaxis	18	119	15.12605042	3.81E-06	0.01100219
GO:0060326~cell chemotaxis	12	58	20.68965517	1.03E-05	0.01482853
GO:0002376~immune system process	33	384	8.59375	4.93E-05	0.03558469
GO:0043931~ossification involved in bone maturation	5	7	71.42857143	4.66E-05	0.03558469
GO:0043406~positive regulation of MAP kinase activity	10	51	19.60784314	8.29E-05	0.04786246

Table S2: List of mouse strains

Strain	Source	Identifier
C57BL/6J CD45.1	University of Edinburgh	
C57BL/6J CD45.2^{+}	University of Edinburgh	
C57BL/6J CD45.1/.2^{+}	University of Edinburgh	
Rag1$^{-/-}$ (*Tgfbr1*$^{fl/fl}$)	University of Glasgow, UK	
Il4ra$^{-/-}$	Prof. Rick Maizels, University of Glasgow, UK	
Lyz2^{Cre}.*Egr2*$^{fl/fl}$	Generated for this study.	*Lyz2*^{Cre} mice – *(24)*<br>*Egr2*$^{fl/fl}$ mice – *(25)*
Fcgr1^{Cre} (*B6-Fcgr1*$^{tm2\text{-}Ciphe}$)	Prof. Bernard Malissen, Dr Sandrine Henri, CIML and CIPHE, France	*(13)*
Fcgr1^{Cre}.*Egr2*$^{fl/fl}$	Generated for this study.	
Cx3cr1$^{tm2.1(cre/ERT2)Jung}$	Jackson Laboratories (JAX)	Stock ID: 020940
Rosa26$^{LSL\text{-}tdRFP}$ (*Gt(Rosa)26Sor*^{tm1Hjf})	Elaine Dzierzak, University of Edinburgh	*(70)*
Cx3cr1$^{Cre\text{-}ERT2}$.*Egr2*$^{fl/fl}$.*Rosa26*$^{LSL\text{-}RFP}$	Generated for this study.	*Cx3cr1*$^{Cre\text{-}ERT2}$ mice – *(44)*
Tgfbr2$^{fl/fl}$	Jackson Laboratories (JAX)	Stock ID: 012603
Fcgr1^{Cre}.*Tgfbr2*$^{fl/fl}$	Generated for this study.	

Table S3: List of antibodies and associated reagents

	Clone	Supplier	Cat. Number	RRID
**Flow cytometry**				
Mouse				
Rat monoclonal CD3 Biotin	17A2	Biolegend	100244	AB_2563947
Rat monoclonal CD11a PE-Cy7	I21/7	Biolegend	153108	AB_2716057
Rat monoclonal CD11b APC-Fire750	M1/70	Biolegend	101262	AB_2572122
Rat monoclonal CD11b PE-Cy7	M1/70	Biolegend	101216	AB_312799
Armenian hamster monoclonal CD11c BV785	N418	Biolegend	117336	AB_2565268
Rat monoclonal CD19 Biotin	6D5	Biolegend	115504	AB_313639
Rat monoclonal CD31 PE-Cy7	390	Biolegend	102418	AB_830757
Rat monoclonal CD31 BV421	390	Biolegend	102424	AB_2650892
Mouse monoclonal CD36 SuperBright 660	HM36	eBioscience	63-0362-82	AB_2734971
Rat monoclonla CD45 BV510	30-F11	Biolegend	103138	AB_2563061
Mouse monoclonal CD45.1 FITC	A20	Biolegend	110706	AB_313495
Mouse monoclonal CD45.2 AF700	104	Biolegend	109822	AB_493731
Rat monoclonal CD63 PE	NVG-2	Biolegend	143904	AB_11204430
Mouse monoclonal CD64 PE-Cy7	X54-5/7.1	Biolegend	139314	AB_2563904
Mouse monoclonal CD64 APC	X54-5/7.1	Biolegend	139306	AB_11219391
Mouse monoclonal CD64 BV421	X54-5/7.1	Biolegend	139309	AB_2562694
Rat monoclonal CD90.2 FITC	30-H12	Biolegend	105305	AB_313176
Rat monoclonal CD102 AF647	3C4	Biolegend	105612	AB_2122182
Rat monoclonal CD103 AF488	2E7	Biolegend	121408	AB_535950
Rat monoclonal CD103 AF647	2E7	Biolegend	121410	AB_535952
Rat monoclonal CD106 (VCAM1) AF647	429	Biolegend	105711	AB_493430
Rat monoclonal CD115 APC	AFS98	Biolegend	135510	AB_2085221
Rat monoclonal CD140a (PDGFRa) PE	APA5	BD Bioesceince	526776	AB_2737787
Rat monoclonal CD146 APC	ME-9F1	Biolegend	134712	AB_2563088
Rat monoclonal CD192 (CCR2) PE	SA203G11	Biolegend	150610	AB_2616982
Rat monoclonal CD326 (EpCAM) BV605	G8.8	Biolegend	118227	AB_2563984
Rat monoclonal CD326 (EpCAM) PE	G8.8	Biolegend	118206	AB_1134172
Rat monoclonal CD354 (TREM1) eF660	TR3MBL1	eBioscience	50-3541-82	AB_2574205
Mouse monoclonal C/EBPb AF647	H7	Santa Cruz Biotech	Sc-7962	AB_626772
Mouse monoclonal CX3CR1 AF647	SA011F11	Biolegend	149004	AB_2564273
Rat monoclonal EGR2 PE	erongr2	eBioscience	12-6691-82	AB_10717804
Rat monoclonal EGR2 APC	erongr2	eBioscience	17-6691-82	AB_11151502
Rat monoclonal F4/80 PE	BM8	Biolegend	123110	AB_893486
Rat monoclonal GP38 PE-Cy7	8.1.1	Biolegend	127412	AB_10613648
Recombinant Ki67 FITC	REA183	Miltenyi Biotec	130-117-691	AB_2733585
Rat monoclonal Ly6C eFluor450	HK1.4	eBioscience	48-5932-82	AB_10805519
Rat monoclonal Ly6C PerCP-Cy5.5	HK1.4	Biolegend	128012	AB_1659241
Rat monoclonal Ly6G Biotin	1A8	Biolegend	127604	AB_1186108
Rat monoclonal Lyve1 eFluor 660	ALY7	eBioscience	50-0443-82	AB_10597449
Rat monoclonal MerTK PE	2B10C42	Biolegend	151506	AB_2617037
Rat monoclonal MHCII (IA-IE) AF700	M5/114.15.2	Biolegend	107622	AB_493727
Mouse monoclonal NK1.1 Biotin	PK136	Biolegend	108704	AB_313391
Rat monoclonal SiglecF PE-CF594	E50-2440	BD Bioscience	562757	AB_2687994
Rat monoclonal Siglec H PE	551	Biolegend	129605	AB_1227763
Human				
Rabbit polyclonal EGR2 (unconjugated)		Invitrogen	PA565091	AB_2662529
Mouse monoclonal HLA-DR eFluor450	LN3	eBioscience	48-9956-42	AB_10718248
Mouse monoclonal CD3 FITC	UCHT1	Biolegend	300452	AB_2564148
Mouse monoclonal CD19 FITC	HIB19	Biolegend	302206	AB_314236
Mouse monoclonal CD56 FITC	5.1H11	Biolegend	362546	AB_2565964
Mouse monoclonal CD66b FITC	G10F5	Biolegend	305104	AB_314496
Mouse monoclonal CD163 APC	RM3/1	Biolegend	326510	AB_2564015
Rat monoclonal CD11b PE-Cy7	M1/70	Biolegend	101216	AB_312799
Other flow cytometry reagents				
Streptavidin BV650		Biolegend	405232	
7-AAD	N/A	Biolegend	420404	
Zombie NIR Fiable Viability Dye		Biolegend	423106	
**Immunofluorescence imaging**				
Rabbit polyclonal CD68 (unconjugated)	N/A	Abcam	Ab125212	AB_10975465
Mouse monoclonal aSMA Cy3	1A4	Merck (Sigma)	C6198	AB_476856
Donkey anti-rabbit AF488	N/A	ThermoFisher	A-21206	AB_2535792
Ly6G-Dylight550	1A8	Novusbio	FAB10371L	

Rat monoclonal CD31 BV421	390	Biolegend	102424	AB_2650892
Rat monoclonal CD11b AF647	M1/70	Biolegend	101218	AB_389327
Armenian hamster monoclonal CD11c AF594	N418	Biolegend	117346	AB_2563323
Rat monoclonal CD45-Spark-NIR	30-F11	Biolegend	103168	AB_2832301

Table S4: List of primers

	Forward	Reverse
Ppia	5’-ACGCCACTGTCGCTTTTC-3	5’-CTGCAAACAGCTCGAAGGA-3’
Car4	5’-CAAACCAAGGATCCTAGAAGCA-3’	5’-GGGGACTGCTGATTCTCCTT-3’
Fabp1	5’-CCATGACTGGGGAAAAAGTC-3’	5’-GCCTTTGAAAGTTGTCACCAT-3’
Col3a1	5’-AACCTGGTTTC TTCTCACCCTTC-3’	5’-ACTCATAGGACTGACCAAGGTGG-3’
Pdgfrb	5’-TCCAGGAGTGATACCAGCTTT-3’	5’-CAGGAGCCATAACACGGACA-3’
